# Kidney Injury Evoked by Fine Particulate Matter: Risk Factor, Causation, Mechanism and Intervention Study

**DOI:** 10.1002/advs.202403222

**Published:** 2024-09-24

**Authors:** Tong Hou, Yuqing Jiang, Jiyang Zhang, Renjie Hu, Sanduo Li, Wenjun Fan, Rucheng Chen, Lu Zhang, Ran Li, Li Qin, Weijia Gu, Yue Wu, Lina Zhang, Xiang Zeng, Qinghua Sun, Yingying Mao, Cuiqing Liu

**Affiliations:** ^1^ School of Public Health Zhejiang Chinese Medical University Hangzhou China; ^2^ Zhejiang International Science and Technology Cooperation Base of Air Pollution and Health Hangzhou China

**Keywords:** fine particulate matter, kidney injury, oxidative stress, autophagy, pyroptosis

## Abstract

Fine particulate matter (PM_2.5_) is suggested to pose a severe risk to the kidneys by inducing functional degradation and chronic kidney diseases (CKD). This study aims to explore the nephrotoxicity of PM_2.5_ exposure and the underlying mechanism. Herein, based on the UK Biobank, it is found that per interquartile range (IQR) increase in PM_2.5_ is associated with a 6% (95% CI: 1%–11%), 7% (95% CI: 3%–11%), 9% (95% CI: 4%–13%), 11% (95% CI: 9%–13%), and 10% (95% CI: 8%–12%) increase in the risk of nephritis, hydronephrosis, kidney stone, acute renal failure, and CKD, respectively. In experimental study, noticeable kidney injury, which is the initiation of kidney diseases, is observed with PM_2.5_ exposure in C57BL/6N mice (*n* = 8), accompanied with oxidative stress, autophagy and pyroptosis. In vitro, HK‐2 cells with PM_2.5_‐stimulation exhibit tubulopathy, increased reactive oxygen species (ROS) generation and activated pyroptosis and autophagy. All changes are abolished by ROS scavenger of N‐acetyl‐L‐cysteine (NAC) both in vivo and in vitro. In conclusion, the study provides evidence showing that PM_2.5_ exposure is associated with 5 kinds of kidney diseases by directly inducing nephrotoxicity, in which ROS may be the potential target by triggering autophagy and pyroptosis.

## Graphical abstract

PM_2.5_ is associated with increased risk of 5 kinds of kidney diseases and is a direct evoker of kidney injury. Additionally, redox imbalance is the key pathologic event in kidney injury which evoked mitochondrial damage and activated autophagy and pyroptosis. Further strategies for ROS intervention and efforts to improve air quality may ease the burden of kidney diseases.

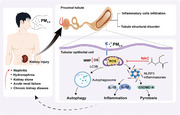

## 1 Introduction

Particulate matter (PM) has become a global public health concern in recent decades. Fine PM (PM_2.5_; aerodynamic diameter <2.5 µm) could deeply penetrate small airways and alveoli and therefore exert toxicological effects or induce diseases of the lung and extrapulmonary organs, including cardiovascular disease, brain disorders, diabetes mellitus, and cancer.^[^ ^1^ ^]^ Observational epidemiological studies suggest that regional PM_2.5_ exposure may impair renal function and aggravate chronic kidney disease (CKD) process over time.^[^ ^2^ ^]^ However, the association of PM_2.5_ exposure with other renal diseases is yet to be understood. Additionally, investigations on the nephrotoxicity of PM_2.5_ exposure have not been performed yet.

Kidney injury is an early event in kidney diseases and is evidenced by structural and functional abnormalities.^[^ ^3^ ^]^ Elucidating the exact histological and functional alterations in the kidney is an effective way to identify the cause (PM_2.5_ exposure) and effect (kidney injury). Renal tubules, as a significant portion of the kidney, are susceptible to various injuries, including toxins, hypoxia, metabolic imbalance and even senescence.^[^ ^4^ ^]^ Emerging evidence reveals that tubulopathy especially proximal tubule lesions will exacerbate the occurrence and progression of kidney diseases.^[^ ^5^ ^]^ Tubular epithelial cells (TECs) have been demonstrated to be important units in renal repair and CKD progression. TECs have innate immune properties, producing and releasing bioactive mediators in response to physical injury, and further drive interstitial inflammation and fibrosis. Undoubtedly, kidney inflammation is the early and important cause of kidney diseases, as evidenced by the aggregation of immunocytes, fluid, and extracellular matrix surrounding the interstitium, as well as inflammatory cell infiltration into tubular cells.^[^ ^6^ ^]^ Thus, clarifying the PM_2.5_‐impaired TECs and the underlying molecular mechanisms is necessary.

Autophagy and pyroptosis are two different types of programmed cell death (PCD). Autophagy is the predominant cellular process in which cytoplasmic substrates are delivered to lysosomes by vesicle encapsulation for degradation. Autophagy has been found to play a dominant role in a variety of disease models. Besides, autophagy inhibitors have also been reported to eliminate or alleviate the symptoms of cardiovascular and respiratory diseases induced by PM_2.5_.^[^ ^7^ ^]^ Currently, pyroptosis mediated by gasdermins (GSDMs) is involved in kidney diseases characterized by NOD‐like receptor thermal protein domain‐associated protein 3 (NLRP3) inflammasome, membrane pore formation, and cytokine release.^[^ ^8^ ^]^ Suppressing NLRP3‐mediated pyroptosis can ameliorate acute kidney injury, whereas NLRP3 inflammasome activation promotes diabetic kidney progression.^[^ ^9^ ^]^ Based on the above evidence, we hypothesized that autophagy and pyroptosis may be critically involved in PM_2.5_‐induced kidney injury.

Oxidative stress (OS) is a protective mechanism that occurs in the body after experiencing abnormal damage, and its main features include excessive production of reactive oxygen species (ROS) and insufficient antioxidant capacity.^[^ ^10^ ^]^ In addition to immunocytes, epithelial cells release ROS in response to PM_2.5_ stimulation, which simultaneously produces inflammatory cytokines.^[^ ^11^ ^]^ Excessive ROS accumulation impairs mitochondrial function and morphology, leading to decreased mitochondrial membrane potential (MMP) and altered permeability, resulting in various PCDs such as autophagy and pyroptosis.^[^ ^12^ ^]^ Additionally, ROS generated by impaired mitochondria leads to imbalance of intracellular redox metabolism, producing increased ROS by feedback, and eventually contributing to damage to the mitochondria or other organelles.^[^ ^13^ ^]^ OS may directly or indirectly contribute to acute kidney injury and CKD by stimulating autophagy and pyroptosis.^[^ ^14^ ^]^ Therefore, we speculated that OS‐evoked autophagy and pyroptosis may be the critical molecular mechanisms underlying PM_2.5_‐regulated nephrotoxicity. Accordingly, the timely elimination of excessive ROS may attenuate PM_2.5_‐induced kidney injury by rectifying autophagy and pyroptosis.

Thus, we investigated the correlation between PM_2.5_ exposure and kidney diseases through a prospective cohort study, identified the causation between PM_2.5_ exposure and kidney injury and the early event of kidney diseases in animal studies, and explored the underlying mechanism and potential intervention under in vivo and in vitro conditions.

## 2 Results

### 2.1 High PM_2.5_ Exposure Levels were Associated with Increased Risks of Kidney Diseases

During a median follow‐up of 12 years (interquartile range (IQR): 11.3–12.7), 2060 nephritis, 3384 hydronephrosis, 2758 kidney stone, 14 643 acute renal failure, 13 500 chronic kidney disease and 1320 kidney cancer events were documented in the analytical cohort. **Table** **1** presents the baseline characteristic distribution of the study participants according to low and high PM_2.5_ exposure levels. Briefly, the percentages of incident cases of nephritis (*p* < 0.001), hydronephrosis (*p =* 0.004), kidney stones (*p* < 0.001), acute renal failure (*p* < 0.001) and CKD (*p* < 0.001) were higher in participants with high PM_2.5_ exposure levels.

**Table 1:** Population characteristics included in the study.

Characteristic	Low PM_2.5_ pollution^a)^(*n* = 236 474)	High PM_2.5_ pollution^a)^(*n* = 211 611)	*p*‐value
Sex, %			
Male	54.8(*n* = 129 513)	54.3(*n* = 114 968)	0.003
Female	45.2(*n* = 106 961)	45.7(*n* = 96 643)	
Age, y, mean (SD)	57.00(7.96)	55.98(8.22)	<0.001
Age, %			
<60 years	54.3(*n* = 128 296)	59.4(*n* = 125 699)	<0.001
≥60 years	45.7(*n* = 108 178)	40.6(*n* = 85 912)	
Race/ethnicity, n. (%)			
White	96.4(*n* = 227 929)	92.1(*n* = 194 810)	<0.001
Others	3.6(*n* = 8545)	7.9(*n* = 16 801)	
BMI, kg m^−2^, mean (SD)	27.25(4.61)	27.61(4.97)	<0.001
BMI, %			
Underweight (<18.5 kg m^−2^)	0.5(*n* = 1087)	0.6(*n* = 1241)	<0.001
Normal (18.5–24.9 kg m^−2^)	33.3(*n* = 78 657)	31.8(*n* = 67 367)	
Overweight (25–29.9 kg m^−2^)	43.4(*n* = 102 542)	41.6(*n* = 87 952)	
Obesity (≥30 kg m^−2^)	22.9(*n* = 54 188)	26.0(*n* = 55 051)	
Education level, %			
College or university degree	38.6(*n* = 91 254)	39.3(*n* = 83 076)	<0.001
Professional education	54.9(*n* = 129 909)	54.7(*n* = 115 774)	
Other	6.5(*n* = 15 311)	6.1(*n* = 12 761)	
Household income, £, %			
Less than 18 000	19.6(*n* = 46 402)	26.5(*n* = 56 023)	<0.001
18 000 to 31 000	25.3(*n* = 59 890)	25.8(*n* = 54 598)	
31 000 to 52 000	27.0(*n* = 63 906)	24.9(*n* = 52 736)	
52 000 to 100 000	22.1(*n* = 52 314)	18.1(*n* = 38 283)	
Greater than 100 000	5.9(*n* = 13 962)	4.7(*n* = 9971)	
Smoking status, %			
Never	56.8(*n* = 134 409)	52.6(*n* = 111 367)	<0.001
Previous	34.7(*n* = 82 017)	34.8(*n* = 73 608)	
Current	8.5(*n* = 20 048)	12.6(*n* = 26 636)	
Drinking status, %			
Never	3.8(*n* = 8873)	5.2(*n* = 11 057)	<0.001
Previous	3.0(*n* = 7090)	4.1(*n* = 8728)	
Current	93.2(*n* = 220 511)	90.7(*n* = 191 826)	
Physical activity, %			
Yes	73.1(*n* = 172 873)	70.1(*n* = 148 687)	<0.001
No	26.9(*n* = 63 601)	29.9(*n* = 62 924)	
Systolic blood pressure (SBP; mmHg)^b)^	140.30(19.67)	139.07(19.67)	<0.001
Glycated hemoglobin (HbA1c; mmol mol^−1^)^b)^	35.94(6.42)	36.23(6.93)	<0.001
Nephritis cases, %	0.5(*n* = 1149)	0.6(*n* = 1231)	<0.001
Hydronephrosis cases, %	0.8(*n* = 1931)	0.9(*n* = 1896)	0.004
Kidney stone cases, %	0.7(*n* = 1559)	0.8(*n* = 1605)	<0.001
Acute renal failure cases, %	3.5(*n* = 8203)	4.2(*n* = 8803)	<0.001
Chronic kidney disease, %	3.2(*n* = 7585)	3.8(*n* = 7944)	<0.001
Kidney cancer, %	0.3(*n* = 798)	0.3(*n* = 688)	0.49

Definition of abbreviations: PM_2.5_ = particulate matter <2.5µm;

^a)^ Defined by WHO guideline value of PM_2.5_: low (<10 µg/m^3^) and high (>10 µg/m^3^);

^b)^ Mean and standard deviation.

After adjusting for potential confounders, PM_2.5_ exposure was associated with increased risk of kidney diseases (**Table** **2**). Specifically, per IQR increase in PM_2.5_ was associated with a 6% (95% CI: 1%–11%), 7% (95% CI: 3%–11%), 9% (95% CI: 4%–13%), 11% (95% CI: 9%–13%) and 10% (95% CI: 8%–12%) increase in nephritis, hydronephrosis, kidney stone, acute renal failure, and CKD, respectively. However, the association with kidney cancer was statistically insignificant (*p* = 0.369).

**Table 2:** Associations of exposure to PM_2.5_ with the risk of kidney diseases.

Kidney diseases	Incidence/Person‐years	Model 1		Model 2^b)^		Model 3^c)^	
		HR (95%CI)	*p* value	HR (95%CI)	*p* value	HR (95%CI)	*p* value
Nephritis							
PM_2.5_ pollution(µg m^−3^)	2380/5 268 209	1.09(1.05–1.13)	7.51 × 10^−6^	1.10(1.06–1.14)	3.52 × 10^−7^	1.04(1.01–1.08)	0.026414
PM_2.5_ pollution (per IQR)	2380/5 268 209	1.11(1.06–1.17)	7.51 × 10^−6^	1.13(1.08–1.18)	3.52 × 10^−7^	1.06(1.01–1.11)	0.026414
Low PM_2.5_ pollution^a)^	1149/2 778 058	Reference	Reference	Reference	Reference	Reference	Reference
High PM_2.5_ pollution^a)^	1231/2 490 151	1.20(1.10–1.30)	1.35 × 10^−5^	1.22(1.13–1.33)	9.55 × 10^−7^	1.11(1.03–1.21)	0.010008
Hydronephrosis							
PM_2.5_ pollution(µg m^−3^)	3827/5 269 720	1.05(1.02–1.08)	0.00121	1.08(1.05–1.11)	6.73 × 10^−7^	1.05(1.02–1.08)	0.000854
PM_2.5_ pollution (per IQR)	3827/5 269 720	1.06(1.02–1.10)	0.00121	1.10(1.06–1.14)	6.73 × 10^−7^	1.07(1.03–1.11)	0.000854
Low PM_2.5_ pollution^a)^	1931/2 778 731	Reference	Reference	Reference	Reference	Reference	Reference
High PM_2.5_ pollution^a)^	1896/2 490 989	1.09(1.03–1.16)	0.006	1.15(1.08–1.22)	2.23 × 10^−5^	1.10(1.03–1.17)	0.004
Kidney stone							
PM_2.5_ pollution(µg m^−3^)	3164/5 267 353	1.09(1.06–1.13)	2.04 × 10^−8^	1.10(1.07–1.14)	2.93 × 10^−9^	1.07(1.03–1.10)	7.04 × 10^−5^
PM_2.5_ pollution (per IQR)	3164/5 267 353	1.12(1.08–1.17)	2.04 × 10^−8^	1.13(1.09–1.18)	2.93 × 10^−9^	1.09(1.04–1.13)	7.04 × 10^−5^
Low PM_2.5_ pollution^a)^	1559/2 777 759	Reference	Reference	Reference	Reference	Reference	Reference
High PM_2.5_ pollution^a)^	1605/2 489 594	1.15(1.07–1.23)	1.02 × 10^−4^	1.16(1.08–1.25)	2.41 × 10^−5^	1.10(1.02–1.18)	0.00864
Acute renal failure							
PM_2.5_ pollution(µg m^−3^)	17006/5 244 446	1.12(1.10–1.13)	<2 × 10^−16^	1.15(1.14–1.17)	<2 × 10^−16^	1.08(1.07–1.10)	<2 × 10^−16^
PM_2.5_ pollution (per IQR)	17006/5 244 446	1.15(1.13–1.17)	<2 × 10^−16^	1.20(1.18–1.22)	<2 × 10^−16^	1.11(1.09–1.13)	<2 × 10^−16^
Low PM_2.5_ pollution^a)^	8203/2 767 075	Reference	Reference	Reference	Reference	Reference	Reference
High PM_2.5_ pollution^a)^	8803/2 477 370	1.19(1.16–1.23)	<2 × 10^−16^	1.28(1.24–1.32)	<2 × 10^−16^	1.14(1.10–1.17)	4.12 × 10^−16^
Chronic kidney disease							
PM_2.5_ pollution(µg m^−3^)	15529/5 261 639	1.09(1.08–1.11)	<2 × 10^−16^	1.14(1.12–1.15)	<2 × 10^−16^	1.08(1.06–1.09)	<2 × 10^−16^
PM_2.5_ pollution (per IQR)	15529/5 261 639	1.12(1.10–1.14)	<2 × 10^−16^	1.18(1.15–1.20)	<2 × 10^−16^	1.10(1.08–1.12)	<2 × 10^−16^
Low PM_2.5_ pollution^a)^	7585/2 776 379	Reference	Reference	Reference	Reference	Reference	Reference
High PM_2.5_ pollution^a)^	7944/2 485 260	1.17(1.13–1.21)	<2 × 10^−16^	1.26(1.22–1.30)	<2 × 10^−16^	1.14(1.11–1.18)	<2 × 10^−16^
Kidney cancer							
PM_2.5_ pollution(µg m^−3^)	1486/5 277 971	0.97(0.92–1.02)	0.215	1.01(0.96–1.06)	0.722	0.98(0.93–1.03)	0.369137
PM_2.5_ pollution (per IQR)	1486/5 277 971	0.96(0.90–1.02)	0.215	1.01(0.95–1.08)	0.722	0.97(0.91–1.03)	0.369137
Low PM_2.5_ pollution^a)^	798/2 782 543	Reference	Reference	Reference	Reference	Reference	Reference
High PM_2.5_ pollution^a)^	688/2 495 428	0.96(0.87–1.06)	0.433	1.03(0.93–1.14)	0.5396	0.98(0.88–1.08)	0.67772

Definition of abbreviations: PM_2.5_ = particulate matter <2.5 µm; HR = hazard ratio; CI = confidence interval.

^a)^ Defined by WHO guideline value of PM_2.5_: low (<10 µg m^−3^) and high (>10 µg m^−3^).

Model1: crude model

^b)^ Model 2: adjusted for age, sex and ethnicity.

^c)^ Model 3: Model 2+education level, average total household income before tax, physical activity, smoking status, drinking status, BMI, SBP and HbA1c.

Similarly, compared with individuals exposed to low PM_2.5_ concentrations, those exposed to high PM_2.5_ levels showed increased risk of nephritis (HR: 1.11; 95% CI: 1.03–1.21), hydronephrosis (HR: 1.10; 95% CI: 1.03–1.17), kidney stone (HR: 1.10; 95% CI: 1.02–1.18), acute renal failure (HR: 1.14; 95% CI: 1.10–1.17) and CKD (HR: 1.14; 95% CI: 1.11–1.18) (Table 2).

The potential non‐linear relationships of exposure to PM_2.5_, with the risk of each kidney disease, were further assessed using restricted cubic spline models. Non‐linearity was not detected for the associations between PM_2.5_, nephritis (*P* non‐linear = 0.052), hydronephrosis (*P* non‐linear = 0.505), kidney stones (*P* non‐linear = 0.957), CKD (*P* non‐linear = 0.289) or kidney cancer (*P* non‐linear = 0.230) (Figure S2, Supporting Information). However, non‐linear correlations were found between exposure to PM_2.5_ and the risk of acute renal failure (*P* non‐linear = 0.012). In the sensitivity analyses using non‐imputed data, the associations between exposure to PM_2.5_ and the risk of kidney diseases remained consistent (Tables S2 and S3, Supporting Information).

### 2.2 PM_2.5_ Exposure Induced Renal Injury

**Figure 1:**
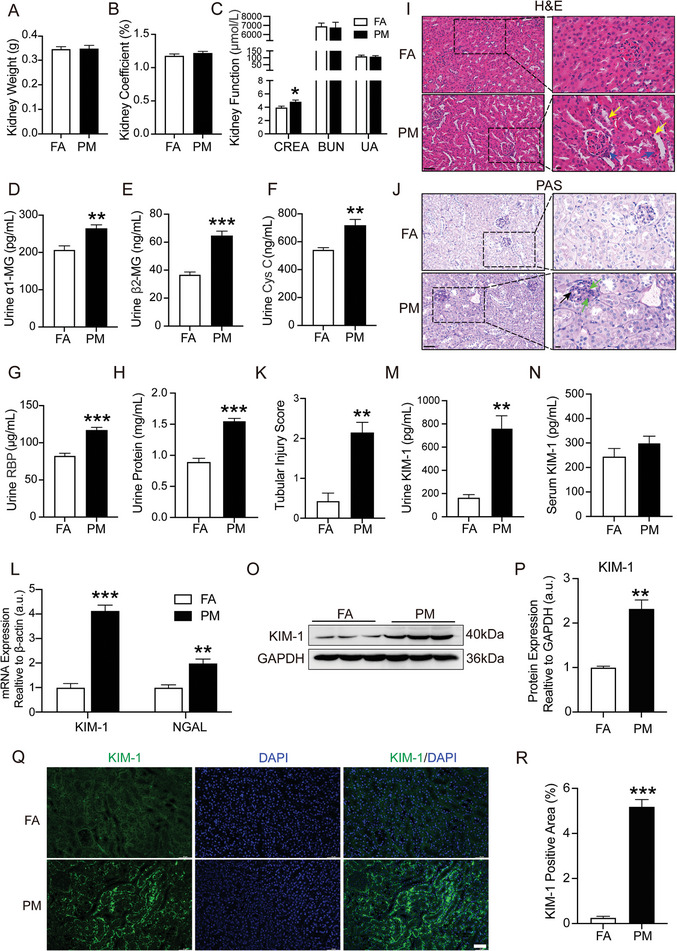
PM_2.5_ exposure induced kidney injury. A) Kidney weight. B) Kidney Coefficient. C) Serum level of CREA, BUN and UA in mice. D) Urine α1‐MG. E) Urine β2‐MG. F) Urine Cys C. G) Urine RBP. H) Urine Protein. I–K) Representative micrographs of renal HE stains, PAS staining and quantification analysis in different groups. Scale bar = 50 µm. Locally enlarged images of kidney tissue sections (scale bar = 20 µm). Yellow arrows mark Dilated renal tubules, blue arrows mark increased inflammatory cells, green arrows mark thylakoid hyperplasia, black arrows mark basement membrane thickening. L) Gene expression of KIM‐1 and NGAL in renal tissues was quantitated using qRT‐PCR. M,N) KIM‐1 levels in urine and serum. O,P) Western blot for renal KIM‐1 protein and quantification analysis. Q,R) Immunofluorescence staining images of KIM‐1 expression in renal sections and quantification analysis. (*n* = 8) (*p < 0.05, **p < 0.01 and ***p < 0.001 versus FA.).

The association of PM_2.5_ exposure with nephrotoxicity in humans was confirmed with PM_2.5_‐exposed mice by evaluating kidney weight, function and morphology. The differences in the body weight (data not shown), kidney weight (**Figure** **1**A) or kidney organ coefficient (Figure 1B) between groups were lacking. However, CERA increased in response to an 8‐week PM_2.5_ exposure, whereas the changes in BUN or UA were insignificant (Figure 1C). Moreover, tubular function markers, including α1‐microglobulin (α1‐MG), β2‐microglobulin (β2‐MG), cystatin C (Cys C), and retinol‐binding protein (RBP) in the urine were significantly elevated in the urine of PM_2.5_‐exposed mice (Figure 1D–G). Similarly, the urine protein content was higher than that in the FA group (Figure 1H). During PM_2.5_ exposure, the average daily concentration of PM_2.5_ was 90.71 ± 7.99 µg m^−3^. An analysis of the further composition of PM_2.5_ showed that water‐soluble inorganic ions accounted for 45.15%, followed by organic and elemental carbon, which accounted for 33.63% and 7.09%, respectively.^[^ ^15^ ^]^

Histopathological examination of the kidney showed that the structure of the renal tubules of PM_2.5_‐exposed mice was disordered, with apparent inflammatory cell infiltration observed in the periglomerular and renal interstitium (Figure 1I). Additionally, a proliferation of glomerular mesangial cells and a thickened basement membrane were observed (Figure 1J). The tubular injury score significantly increased in response to PM_2.5_ exposure (Figure 1K). Kidney injury molecule 1 (KIM‐1) and neutrophil gelatinase‐associated lipocalin (NGAL) are potential biomarkers for early prediction of kidney damage in clinical practice.^[^ ^16^ ^]^ The mRNA expression of KIM‐1 and NGAL in the kidney statistically increased in response to PM_2.5_ exposure, with a fourfold increase in KIM‐1 levels compared with that in the FA group (Figure 1L). Further examination indicated that urine levels of KIM‐1 were dramatically elevated in response to PM_2.5_ exposure (Figure 1M), although the difference observed in the serum was statistically insignificant (Figure 1N). Immunoblotting and immunofluorescence staining consistently indicated significant increase in KIM‐1 protein level in PM_2.5_‐exposed mice (Figure 1O–R). Taken together, these results indicated that PM_2.5_ exposure induced kidney injury in mice.

### 2.3 Effects of PM_2.5_ Exposure on Inflammation, Pyroptosis and Autophagy in the Kidneys

**Figure 2:**
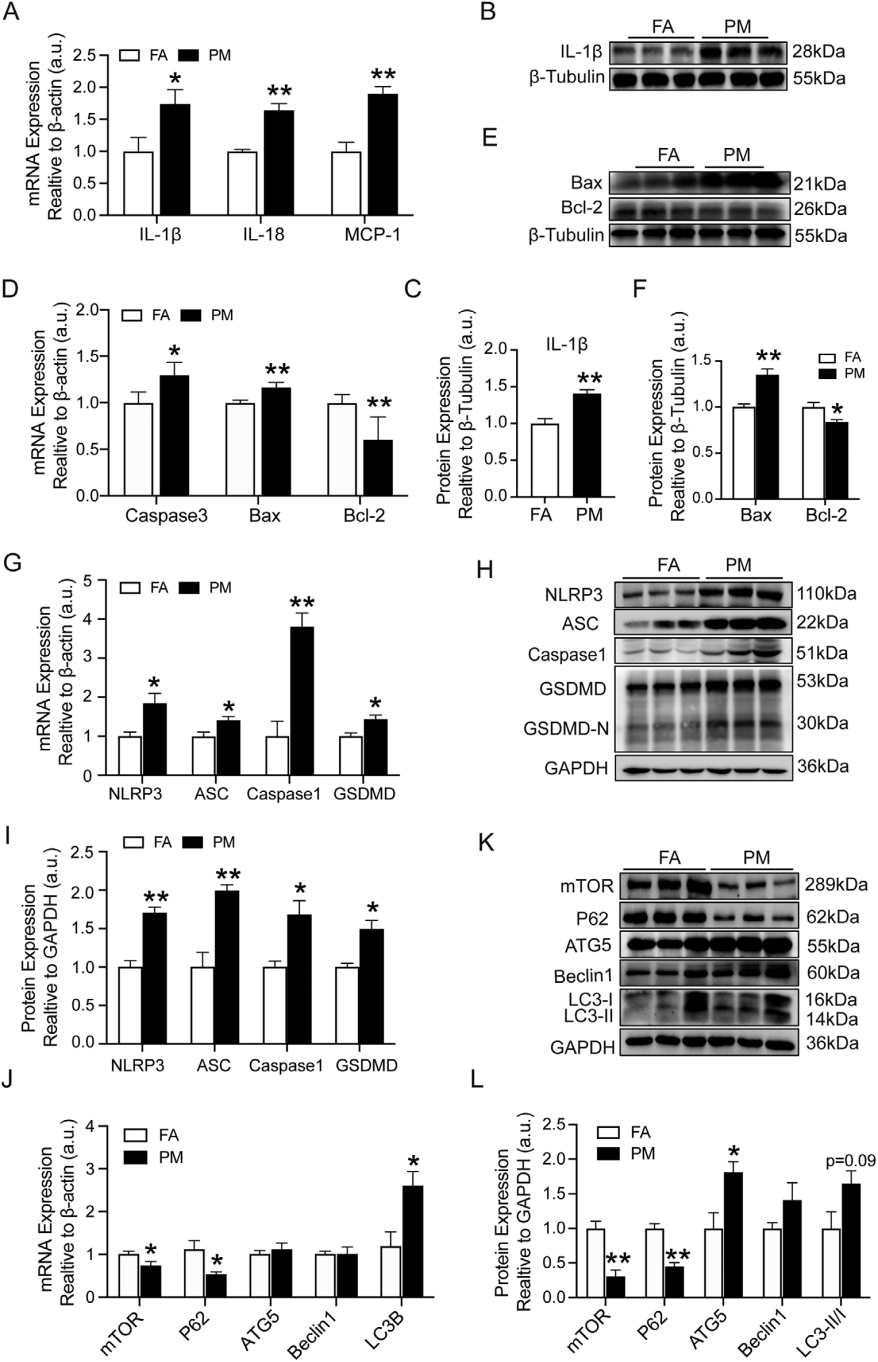
Effects of PM_2.5_ exposure on the inflammation, pyroptosis and autophagy in kidneys. (A) Gene expression of IL‐1β, IL‐18 and MCP‐1 in renal tissues was quantitated using qRT‐PCR. (B, C) Western blot for renal IL‐1β protein and quantification analysis. (D) Gene expression of Caspase3, Bax and Bcl‐2 in renal tissues was quantitated using qRT‐PCR. (E, F) Western blot for renal Bax and Bcl‐2 protein and quantification analysis. (G) Gene expression of NLRP3, ASC, Caspase1 and GSDMD in renal tissues was quantitated using qRT‐PCR. (H, I) Western blot for renal NLRP3, ASC, Caspase1 and GSDMD protein and quantification analysis. (J) Gene expression of mTOR, P62, ATG5, Beclin1 and LC3B in renal tissues was quantitated using qRT‐PCR. (K, L) Western blot for renal mTOR, P62, ATG5, Beclin1 and LC3B protein and quantification analysis. (*n* = 8) (*p < 0.05, **p < 0.01 and ***p < 0.001 versus FA.).

Pathological events in the kidneys in response to PM_2.5_ exposure were examined. mRNA levels of IL‐1β, IL‐18 and MCP‐1 in the kidney were significantly increased in PM_2.5_‐exposed mice (**Figure** **2**A), with IL‐1β elevation being confirmed at protein levels (Figure 2B,C). Additionally, the Caspase3 expression at the mRNA level and Bax at both mRNA and protein levels in mouse kidneys were upregulated compared with that in the FA group. Accordingly, the mRNA and protein expression of renal Bcl‐2 decreased after PM_2.5_ exposure (Figure 2D–F). Next, the NLRP3 inflammasome complex, consisting of NLRP3, apoptosis‐associated speck‐like protein containing CARD (ASC), cysteinyl aspartate‐specific proteinase‐1 (Caspase1), and the pyroptosis gene GSDMD, was examined. Both mRNA and protein levels of these molecules considerably increased in the PM_2.5_‐exposed mice compared with the FA group (Figure 2G–I).

The role of autophagy in PM_2.5_‐induced kidney injury was further evaluated by investigating the mRNA and protein levels of the autophagy‐related molecules mTOR, P62, ATG5, Beclin1 and LC3B. The mRNA levels of mTOR and P62 decreased in the kidney of PM_2.5_‐exposed mice compared to those in the FA group, and the mRNA level of LC3B noticeably increased in the PM_2.5_ group (Figure 2J). Additionally, the protein levels of mTOR and P62 were down‐regulated after PM_2.5_‐treatment, whereas the protein levels of ATG5 and LC3II/LC3I were upregulated (Figure 2K,L). Thus, PM_2.5_ activated pyroptosis and autophagy in the kidneys in vivo.

### 2.4 Effects of PM_2.5_ Exposure on the Tubulopathy, Pyroptosis and Autophagy in the HK‐2 Cells

**Figure 3:**
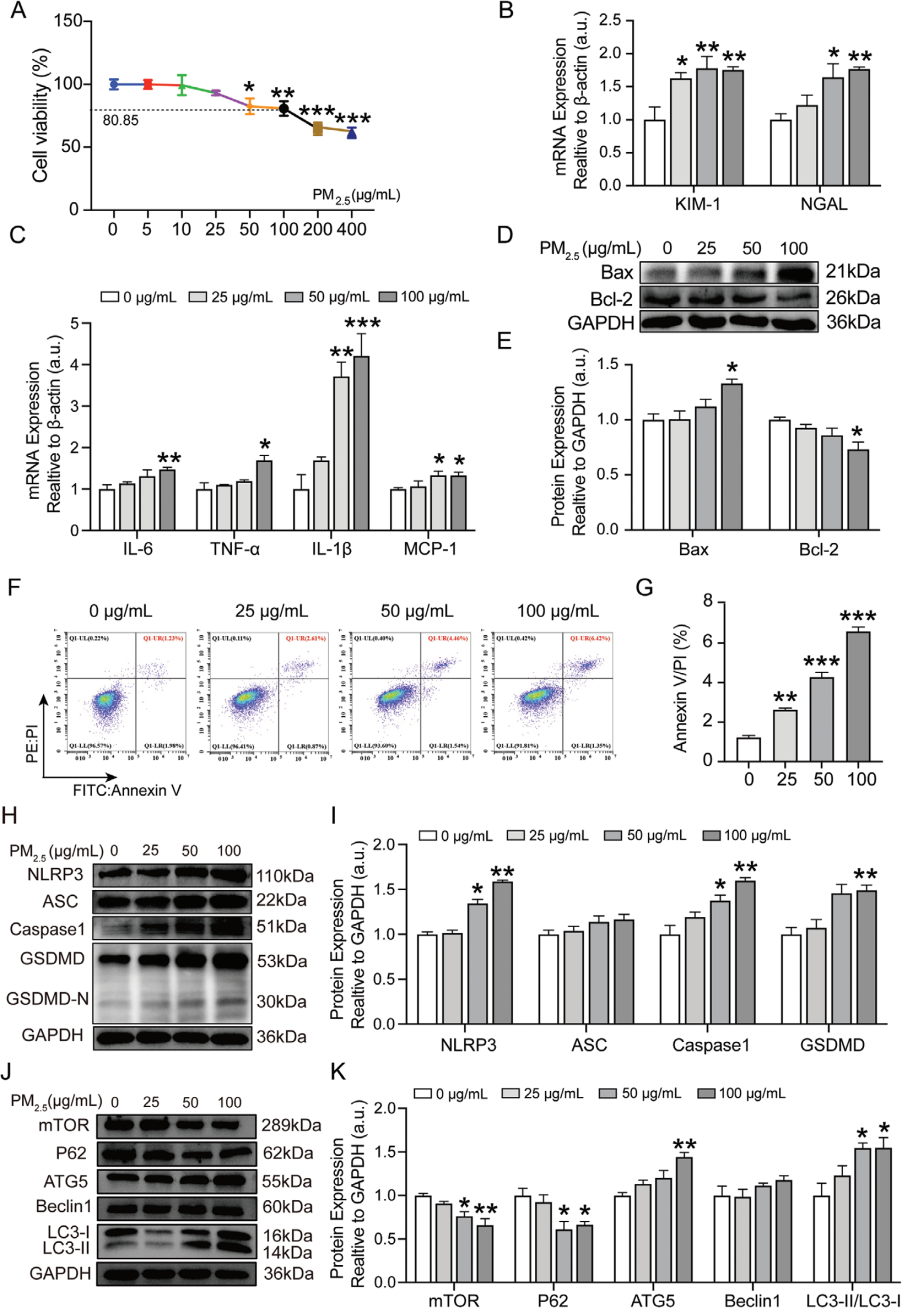
Effects of PM_2.5_ exposure on the tubulopathy, pyroptosis and autophagy in HK‐2 cells. A) Cell viability of HK‐2 cells treated with different concentration of PM_2.5_ (5, 10, 25, 50, 100, 200,400 µg mL^−1^) for 24 h detected by CCK‐8 assay. B) The gene expression of KIM‐1 and NGAL in HK‐2 cells was quantitated using qRT‐PCR. C) The gene expression of IL‐6, TNF‐α, IL‐1β and MCP‐1 in HK‐2 cells was quantitated using qRT‐PCR. D,E) The expression of Bax and Bcl‐2 in HK‐2 cells was analyzed using Western blot and quantification analysis. F) Flow cytometry analysis of apoptosis using Annexin V‐FITC and PI‐PE staining. G) Percentage of apoptosis in each group of HK‐2 cells. H,I) The protein expression of NLRP3, ASC, Caspase1 and GSDMD in HK‐2 cells was analyzed using Western blot and quantification analysis. J,K) The expression of mTOR, P62, ATG5, Beclin1 and LC3B in HK‐2 cells were analyzed using Western blot and quantification analysis. (*n* = 3) (*p < 0.05, **p < 0.01 versus 0 µg mL^−1^).

Subsequently, we confirmed the PM_2.5_‐induced damage by measuring tubulopathy in HK‐2 cells, a human kidney proximal tubular epithelial cell line. The optimum concentration of PM_2.5_ treatment in HK‐2 cells was explored by applying the gradient concentration of PM_2.5_ at 5, 10, 25, 50, 100, 200, and 400 µg mL^−1^ to HK‐2 cells for 24 h and determining the cell viability using the CCK‐8 assay. PM_2.5_ exposure impaired cell viability, with 80.85% at 100 µg mL^−1^ in a dose‐dependent manner (**Figure** **3**A). Therefore, PM_2.5_ concentrations of 25, 50, and 100 µg mL^−1^ were adopted in subsequent experiments. Consistent with the in vivo observations, the expression of KIM‐1 and NGAL was significantly upregulated at the mRNA level in HK‐2 cells in response to PM_2.5_ exposure. KIM‐1 increased at all doses and NGAL increased at higher doses of 50 and 100 µg mL^−1^ (Figure 3B).

PM_2.5_ incubation at 25 µg mL^−1^ induced no inflammation, whereas PM_2.5_ at 50 µg mL^−1^ exerted IL‐1β and MCP‐1 upregulation, and the IL‐6, TNF‐α, IL‐1β and MCP‐1 levels increased at a higher dose of 100 µg mL^−1^ PM_2.5_ in the HK‐2 cells (Figure 3C). Additionally, PM_2.5_‐treatment remarkably upregulated Bax protein and downregulated Bcl‐2 protein at 100 µg mL^−1^ compared to the 0 µg mL^−1^ group (Figure 3D,E). We performed Annexin V/PI using flow cytometry analysis to evaluate cell apoptosis and found that the apoptosis rate of HK‐2 cells increased in response to PM_2.5_ incubation in a dose‐dependent manner (Figure 3F,G).

Pyroptosis and autophagy were examined in PM_2.5_‐treated HK‐2 cells. Consistent with the in vivo experiments, our results revealed a dose‐dependent increase in the pyroptosis‐related proteins including NLRP3, ASC, Caspase1 and GSDMD, which reached a statistical significance at 50 or 100 µg mL^−1^ (Figure 3H,I). Regarding autophagy, the levels of mTOR and P62 proteins were considerably downregulated at PM_2.5_ concentrations of 50 and 100 µg mL^−1^. The ATG5 protein level was upregulated at 100 µg mL^−1^ PM_2.5_ concentration. Additionally, PM_2.5_ stimulation at concentrations of 50 and 100 µg mL^−1^ upregulated the protein levels of LC3II/LC3I (Figure 3J,K).

### 2.5 Effects of PM_2.5_ Exposure on the OS in the Kidneys and HK‐2 Cells

**Figure 4:**
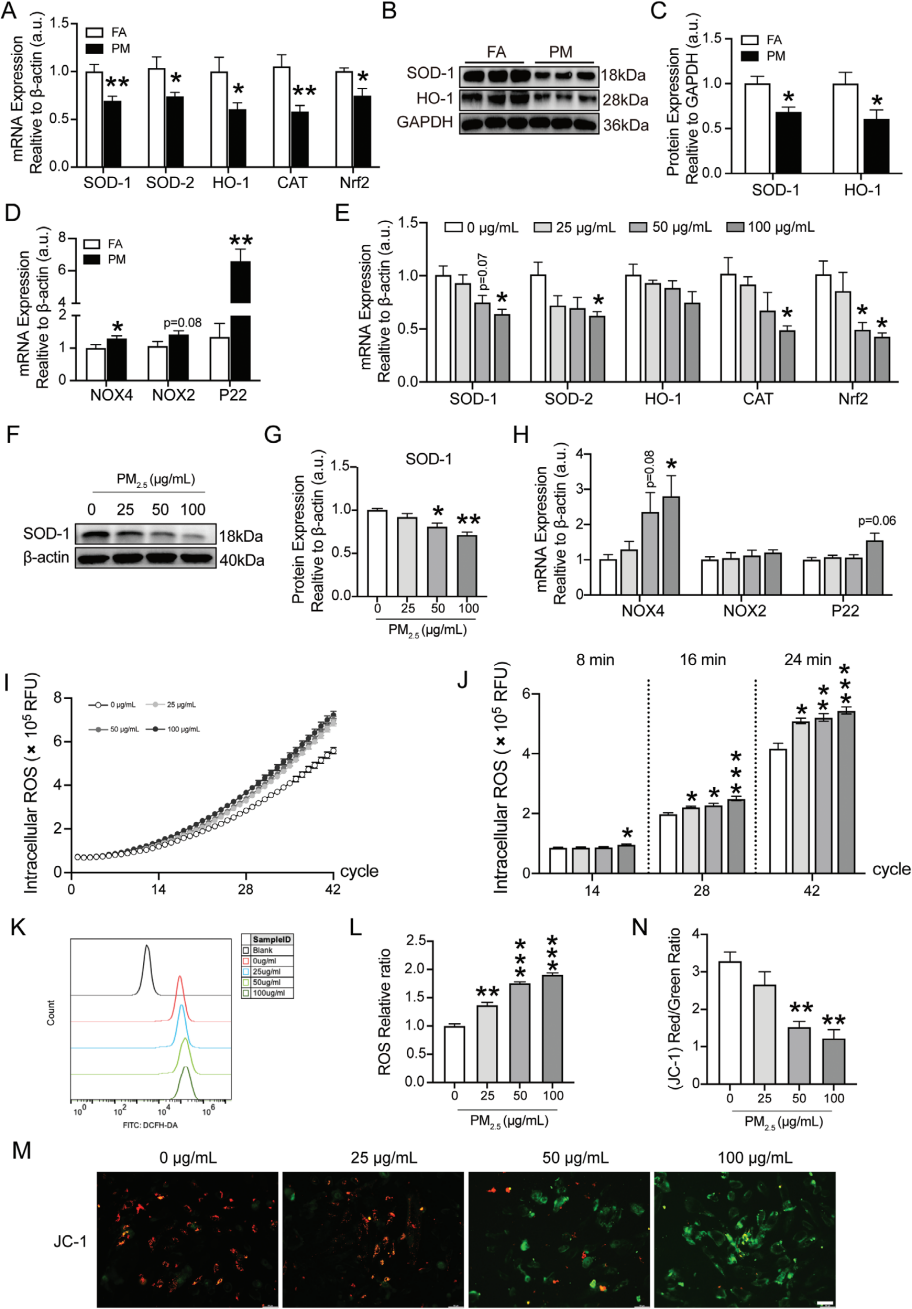
Effects of PM_2.5_ exposure on the oxidative stress in kidneys and HK‐2 cells. A) Gene expression of SOD‐1, SOD‐2, HO‐1, CAT, and Nrf2 in renal tissues was quantitated using qRT‐PCR. B,C) Western blot for renal SOD‐1 and HO‐1 protein and quantification analysis. D) Gene expression of NOX4, NOX2 and P22 in renal tissues was quantitated using qRT‐PCR. (*n* = 8) (*p < 0.05, **p < 0.01 versus FA.) E) Gene expression of SOD‐1, SOD‐2, HO‐1, CAT and Nrf2 in HK‐2 cells was quantitated using qRT‐PCR. F,G) The expression of SOD‐1 in HK‐2 cells was analyzed using Western blot and quantification analysis. H) Gene expression of NOX4, NOX2 and P22 in HK‐2 cells was quantitated using qRT‐PCR. I) To assess the production of intracellular ROS, HK‐2 cells were pre‐incubated with DCFH‐DA for 1 h and subsequently incubated with PM_2.5_ (25, 50, and 100 µg mL^−1^). Fluorescence was measured in real time over 24 min. Data are expressed as relative fluorescence units (RFU). J) RFU data at 8, 16, and 24 min are plotted as histograms of mean ± SEM of ROS production corresponding to 3 independent experiments. K) The ROS levels of HK‐2 cells were determined by flow cytometry. L) ROS relative ratio of HK‐2 cells in each group. M) Representative images of JC‐I staining of HK‐2 cells. Scale bar = 50 µm. N) Red/green fluorescence ratio of JC‐1 staining of HK‐2 cells in each group. (*n* = 3) (*p < 0.05, **p < 0.01, and ***p < 0.001 versus 0 µg mL^−1^).

The mechanism of PM_2.5_‐induced kidney injury was explored by assessing the intracellular redox balance in the kidneys and HK‐2 cells. The levels of antioxidants, including SOD‐1, SOD‐2, HO‐1, CAT and Nrf2, in the kidney of PM_2.5_‐exposed mice were markedly lower than those in the kidney of FA‐exposed mice (**Figure** **4**A). Reduced SOD‐1 and HO‐1 were confirmed at protein levels (Figure 4B,C). Contrastingly, the mRNA levels of oxidants such as NOX4 and P22 in the kidney considerably increased in response to PM_2.5_ exposure (Figure 4D). In addition, mitochondrial function‐related genes, including mitochondrial respiratory chain complex (I, II, III, IV, ATP synthase), mitochondrial dynamics genes (Mfn2, OPA1, Drp1, Fis1), and mitochondrial biosynthesis genes (PGC‐1α), were also detected. Consistently, the expression of mitochondrial respiratory chain complex‐related genes, mitochondrial dynamics ‐related genes and biosynthetic genes was significantly altered in PM group mice compared with controls, indicating impaired mitochondrial function (Figure S3A,B, Supporting Information).

Then effects of PM_2.5_ exposure on the OS balance were examined in HK‐2 cells. The Nrf2 level was considerably downregulated in response to PM_2.5_ exposure at a concentration of 50 µg mL^−1^, and the SOD‐1, SOD‐2, CAT and Nrf2 levels all markedly declined at a higher dose of 100 µg mL^−1^ PM_2.5_ in HK‐2 cells (Figure 4E). Additionally, PM_2.5_ treatment inhibited the protein levels of SOD‐1 in HK‐2 cells in a dose‐dependent manner (Figure 4F,G). Contrastingly, the mRNA levels of NOX4 significantly increased at a PM_2.5_ concentration of 100 µg mL^−1^ (Figure 4H). Accordingly, the activities of SOD and HO‐1 enzymes were assayed in HK‐2 cells. The results showed that SOD and HO‐1 enzyme activities were significantly and dose‐dependently reduced in the PM_2.5_‐treated group compared with the control group (Figure S4A,B, Supporting Information).

Next, the direct effects of PM_2.5_ exposure on ROS were examined (Figure 4I). Notably, increased intracellular ROS at early periods (8 min after PM_2.5_ treatment, the first 14 cycles) was only observed at a higher dose of 100 µg mL^−1^. After 16‐ and 24‐min of PM_2.5_ treatment, the intracellular ROS was considerably elevated in all the doses (25, 50 and 100 µg mL^−1^) (Figure 4J). Furthermore, the elevation of intracellular ROS levels was confirmed using flow cytometry in HK‐2 cells treated with PM_2.5_ for 24 h (Figure 4K,L). MMP of HK‐2 cells was assessed to investigate whether excess ROS production induced by PM_2.5_ exacerbated mitochondrial damage. Fluorescence staining showed that PM_2.5_ treatment decreased the (JC‐1) Red/Green Ratio, a marker of MMP in HK‐2 cells in a dose‐dependent manner (Figure 4M,N). Taken together, these results demonstrated that PM_2.5_ induced a redox imbalance accompanied by mitochondrial damage.

### 2.6 NAC Attenuated the Damage of HK‐2 Cells and the Activation of Autophagy and Pyroptosis Induced by PM_2.5_

**Figure 5:**
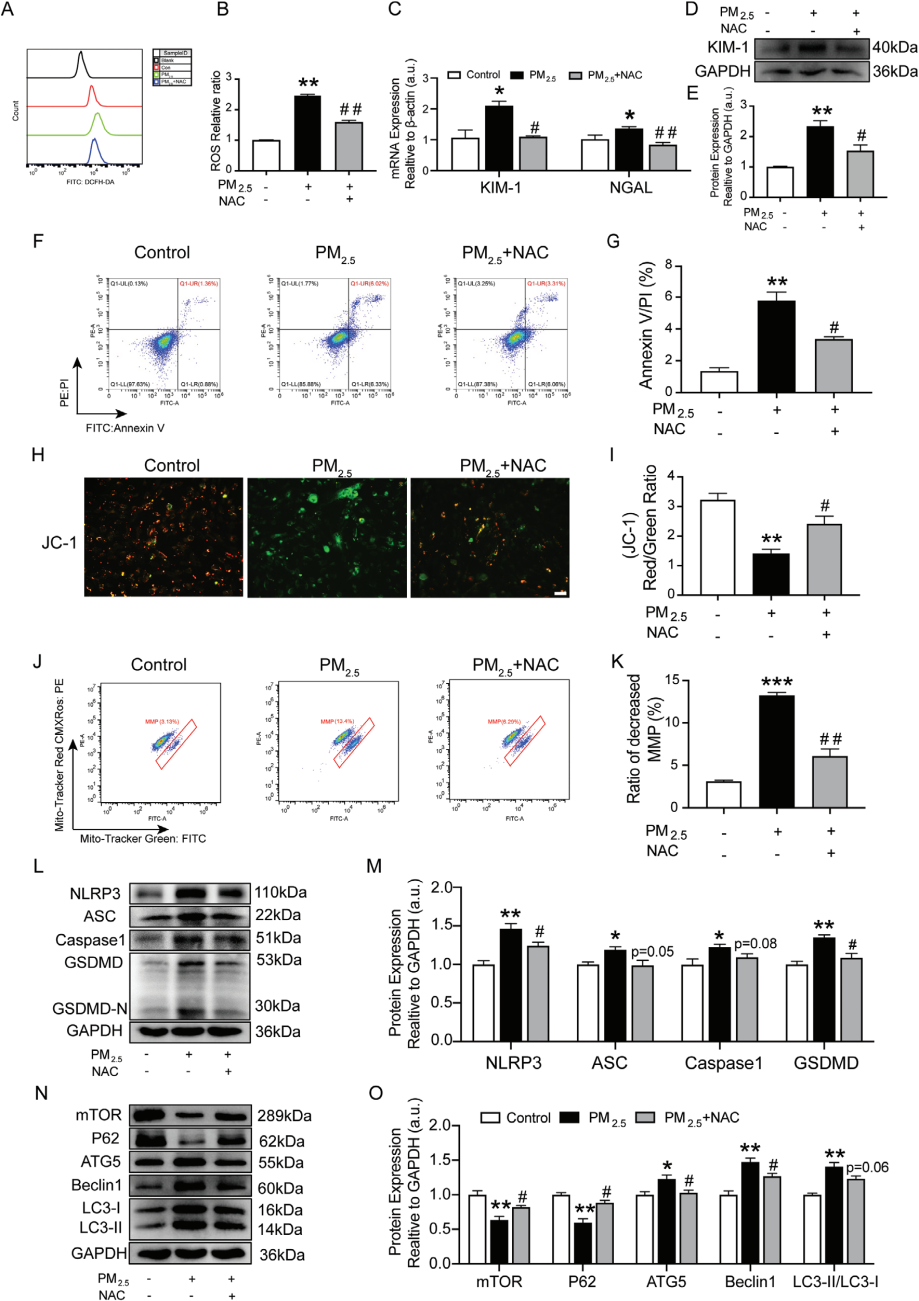
NAC attenuated the damage of HK‐2 cells and the activation of autophagy and pyroptosis induced by PM_2.5_.HK‐2 cells were pre‐treated with 5 mM NAC for 1 h and were then treated with 100 µg mL^−1^ PM_2.5_ for 24 h. A) The ROS levels of HK‐2 cells were determined by flow cytometry. B) ROS relative ratio of HK‐2 cells in each group. C) Gene expression of KIM‐1 and NGAL in HK‐2 cells was quantitated using qRT‐PCR. D,E) Western blot for KIM‐1 protein and quantification analysis. F) Flow cytometry analysis of apoptosis using Annexin V‐FITC and PI‐PE staining. G) Percentage of apoptosis in each group of HK‐2 cells. H) ΔΨm of PM_2.5_‐treated HK‐2 cells were measured by JC‐1 staining under a fluorescence microscope. Scale bar = 50 µm. I) Red/green fluorescence ratio of JC‐1 staining of HK‐2 cells in each group. J) HK‐2 cells were labeled with MitoTracker Red and MitoTracker Green using flow cytometric analysis. K) Flow cytometric analysis of the MMP. L,M) The efficiency of NAC, and its effect on the expression of NLRP3, ASC, Caspase1 and GSDMD in PM_2.5_‐treated HK‐2 cells using Western blot and quantification analysis. N,O) The efficiency of NAC, and its effect on the expression of mTOR, P62, ATG5, Beclin1 and LC3B in PM_2.5_‐treatment HK‐2 cells using Western blot and quantification analysis. (*n* = 3) (*p < 0.05, **p < 0.01 versus Control group. ^#^ *p* < 0.05, ^##^ *p* < 0.01 versus PM_2.5_ group.).

N‐acetyl‐L‐cysteine (NAC), an antioxidant, was used to eliminate ROS. To verify whether PM_2.5_‐induced damage in HK‐2 cells was dependent on ROS, 5 mM NAC was used to scavenge ROS in the HK‐2 cells. Compared to the control group, PM_2.5_‐enhanced ROS production at 100 µg mL^−1^ was partially blocked by NAC pre‐incubation (**Figure** **5**A,B). Simultaneously, PM_2.5_‐upregulated expression of KIM‐1 and NGAL was abolished to different extents by ROS elimination with NAC (Figure 5C–E). Similarly, pre‐incubation with NAC alleviated the PM_2.5_‐increased apoptosis rate in the HK‐2 cells (Figure 5F,G).

Moreover, JC‐1 staining demonstrated the green fluorescence intensity was considerably increased after PM_2.5_ treatment, indicating that the ΔΨ of cells was conspicuously reduced. NAC reversed ΔΨ reduction in PM_2.5_‐exposed cells (Figure 5H,I). Additionally, MitoTracker Green FITC and MitoTracker Red CMXRos were used to measure changes in MMP using flow cytometry. Consistent with the JC‐1 staining, NAC treatment corrected the ratio of decreased MMP as well (Figure 5J,K). Furthermore, NAC pre‐treatment significantly increased the enzyme activity of SOD and decreased the expression level of MDA in HK‐2 cells (Figure S5A,B, Supporting Information). Meanwhile, NAC pre‐treatment also apparently reduced the level of ROS in mitochondria (Figure S5C,D, Supporting Information).

To further verify whether excess ROS was the reason for pyroptosis and autophagy generation in response to PM_2.5_ exposure, a series of molecules were examined under ROS‐scavenging conditions. In response to pre‐incubation with NAC, the levels of pyroptosis markers NLRP3, ASC, Caspase1 and GSDMD in the HK‐2 cells decreased or showed a decreasing trend when compared with the PM_2.5_‐treated group (Figure 5L,M). Regarding autophagy, the inhibited expression of mTOR and P62 and the enhanced expression of ATG5, Beclin1 and LC3II/LC3I in response to PM_2.5_, were blocked by pre‐treatment with NAC in the HK‐2 cells (Figure 5N,O). These findings further proved that scavenging excess ROS blocked pyroptosis and autophagy induced by PM_2.5_ in the HK‐2 cells.

### 2.7 NAC Attenuated PM_2.5_‐Induced Kidney Injury in Mice

**Figure 6:**
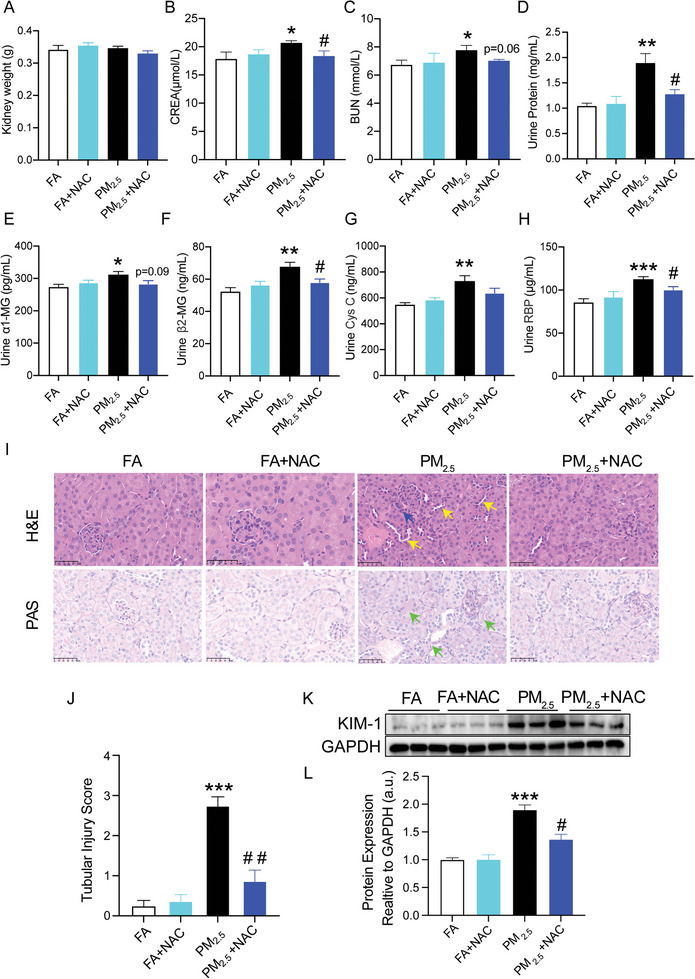
NAC attenuated PM_2.5_‐induced kidney injury in mice. A) Kidney weight. B,C) Serum level of CREA and BUN in mice. D) Urine Protein. E) Urine α1‐MG. F) Urine β2‐MG. G) Urine Cys C. H) Urine RBP. I,J) Representative micrographs of renal HE stains, PAS staining and quantification analysis in different groups. Scale bar = 50 µm. Yellow arrows mark Dilated renal tubules, blue arrows mark increased inflammatory cells, green arrows mark thylakoid hyperplasia, black arrows mark basement membrane thickening. K,L) Western blot for renal KIM‐1 protein and quantification analysis. (*n* = 8) (*p < 0.05, **p < 0.01 and ***p < 0.001 versus FA group. ^#^ *p* < 0.05 versus PM_2.5_ group.).

To further investigate whether NAC could ameliorate PM_2.5_ exposure‐induced renal injury in mice, the indicators of renal weight, function and morphology were assessed in the NAC‐treated mice exposed to PM_2.5_ exposure. There were no differences in body weight (Figure S6A, Supporting Information), kidney weight (**Figure** **6**A), or kidney organ coefficient (Figure S6B, Supporting Information) between the groups. However, mice in NAC‐treated group showed significant decrease in CERA, a tendency to decrease in BUN as well (Figure 6B,C), whereas no significant difference was observed with UA (Figure S6C, Supporting Information). Similarly, the urinary protein content of the NAC‐treated group was significantly lower than the that in mice exposed to PM_2.5_. (Figure 6D). In addition, the elevated levels of β2‐MG and RBP in the urine of PM_2.5_‐exposed mice were significantly attenuated in response to NAC treatment, whereas α1‐MG showed a decreasing tendency in the NAC‐treated mice (Figure 6E–H). Histopathological examination of the kidneys showed improved renal tubular structure with reduced inflammatory cell infiltration and basement membrane thickening in the NAC‐treated mice exposed to PM_2.5_ exposure (Figure 6I). Accordingly, renal tubular injury score was significantly decreased as well (Figure 6J). Meanwhile, protein expression of KIM‐1 in the kidney was significantly inhibited by NAC treatment (Figure 6K,L). These findings further demonstrated that NAC attenuated PM_2.5_‐induced kidney injury in mice.

### 2.8 NAC Attenuated Activation of Autophagy and Pyroptosis Induced by PM_2.5_ in the Kidneys

**Figure 7:**
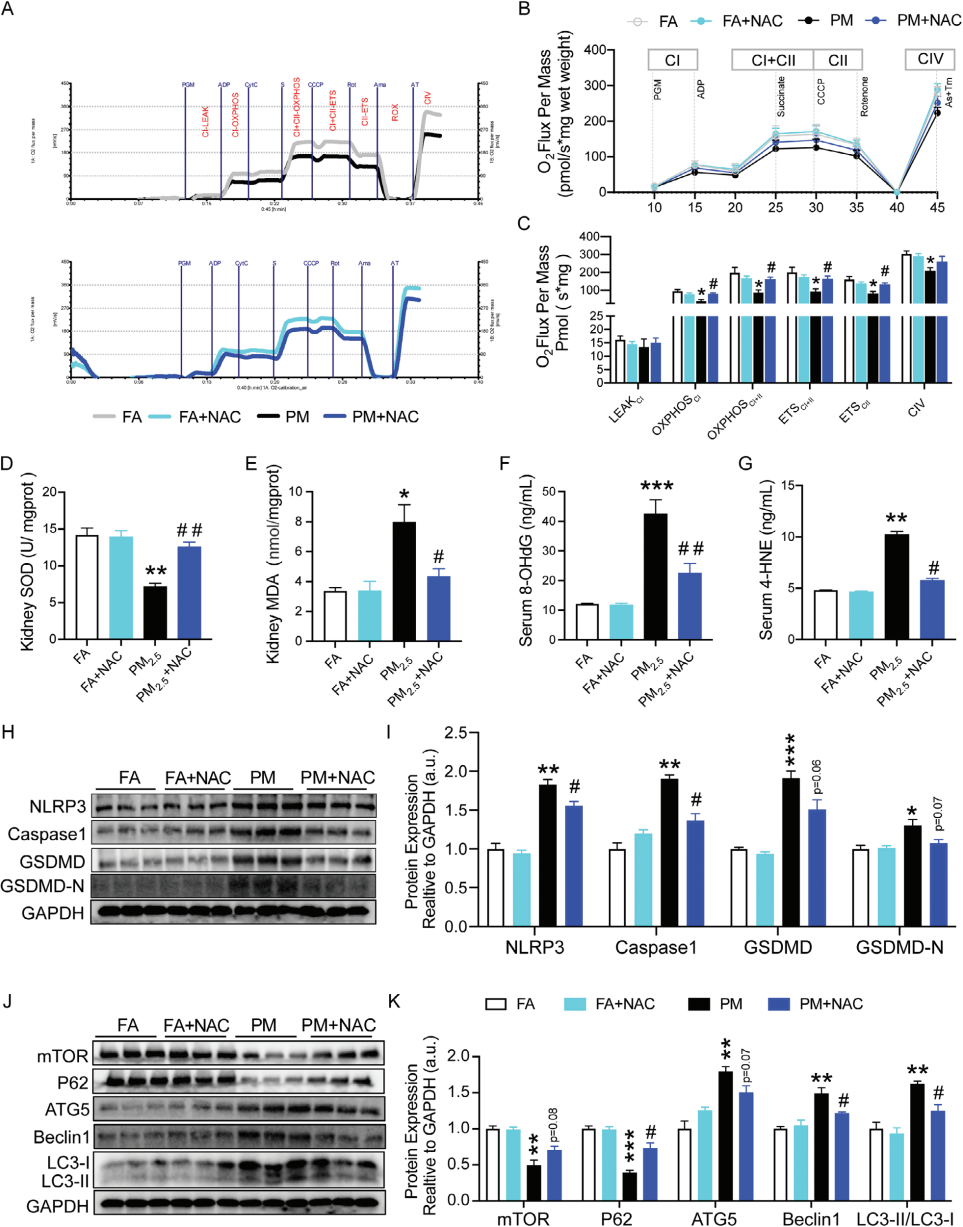
NAC attenuated activation of autophagy and pyroptosis induced by PM_2.5_ in the kidneys. A–C) Representative images and analysis of the mitochondrial oxygen consumption rate (OCR) of the kidney. D,E) Kidney SOD and MDA. F,G) Serum level of 8‐OHdG and 4‐HNE. H,I) Western blot for renal NLRP3, Caspase1 and GSDMD protein and quantification analysis. J,K) Western blot for renal mTOR, P62, ATG5, Beclin1 and LC3B protein and quantification analysis. (*n* = 8) (*p < 0.05, **p < 0.01, and ***p < 0.001 versus FA group. ^#^ *p* < 0.05, ^##^ *p* < 0.01 versus PM_2.5_ group.).

Next, whether excess ROS were responsible for the kidney redox imbalance and activation of autophagy and pyroptosis induced by PM_2.5_ exposure was further elucidated. In PM_2.5_‐exposed mice, mitochondrial oxygen consumption rate was significantly enhanced in kidney tissues in response to NAC treatment (**Figure** **7**A,B), which markedly increased the maximal oxidative phosphorylation capacity and maximal electron transfer capacity of mitochondrial complexes I and II (Figure 7C). The enzyme activity of the antioxidant SOD was significantly increased in the kidneys of NAC‐treated mice exposed to PM_2.5_ (Figure 7D), and consequently the expression of the lipid peroxide MDA was significantly decreased (Figure 7E). Similarly, the PM_2.5_‐increased circulating levels of oxidative DNA damage marker 8‐OHdG and lipid peroxide 4‐HNE were both significantly reduced by NAC treatment (Figure 7F,G). These findings confirmed that NAC indeed inhibited PM_2.5_ exposure‐induced oxidative stress in the kidneys.

Mechanistically, the levels of pyroptosis markers NLRP3, Caspase1 and GSDMD were decreased or showed a trend toward decrease in the kidney of PM_2.5_‐exposed mice treated with NAC (Figure 7H,I). In terms of autophagy, the expression of autophagy markers mTOR and P62 was enhanced, whereas the expression of ATG5, Beclin1 and LC3II/LC3I was reduced in the renal from NAC‐treated mice exposed to PM_2.5_ (Figure 7J,K). These findings further suggested that NAC blocked PM_2.5_‐induced activation of autophagy and pyroptosis in the kidneys.

## 3 Discussion

To our knowledge, it is the first study to assess the nephrotoxicity of PM_2.5_ exposure following a close research frame of “association – causation – mechanism – intervention.” Based on the observational epidemiological investigation, we first verified that PM_2.5_ exposure was associated with increased risk of 5 kinds of kidney diseases. Then, PM_2.5_ exposure was identified as the triggering factor of kidney injury, an early event of kidney diseases, through both in vivo and in vitro experiments. Further, the mechanism may be that exposure to PM_2.5_ jeopardized TEC, which was attributable to redox imbalance‐mediated pyroptosis and autophagy. Finally, NAC treatment attenuated kidney and TEC injury by inhibiting the excessive ROS production.

**Figure 8:**
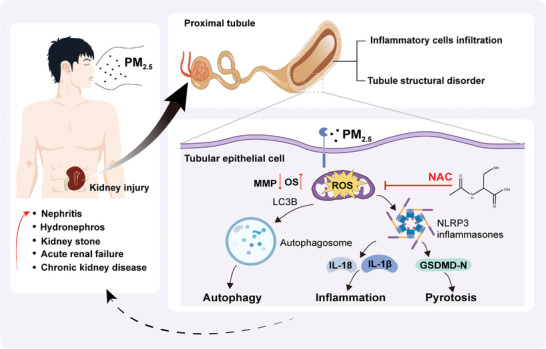
Schematic diagram of mechanisms underlying PM_2.5_‐induced nephrotoxicity.PM_2.5_ exposure is associated with increased risk of kidney diseases. Meanwhile, PM_2.5_ induces the generation of ROS, accompanied with activation of autophagy and pyroptosis, followed by the release of many inflammatory factors, such as IL‐1β and IL‐18, resulting in the occurrence of kidney injury.

The kidney is essential for excretion and endocrine functions. Previous epidemiological studies have shown the association about PM_2.5_ exposure and CKD incidence, suggesting that long‐term exposure to PM_2.5_ increases CKD risk and accelerates progression.^[^ ^17^ ^]^ In our study, in addition to CKD, PM_2.5_ exposure was first identified as a higher risk factor of other kidney diseases, including nephritis, hydronephrosis, kidney stones and acute renal failure. Due to the physiological characteristics of high blood flow, large endothelial surface area and high metabolic activity,^[^ ^18^ ^]^ the kidney is a vulnerable organ to external or internal stimulation. Kidney injury is widely recognized as the initiation of kidney diseases development.^[^ ^19^ ^]^ Thus, elucidating the exact effects of PM_2.5_ exposure on kidney injury will provide important complement to the etiology of kidney diseases.

Based on the analysis of risk factor from epidemiological investigation, the most important finding of the study was that we identified PM_2.5_ as the direct inducing factor of kidney injury in mice using whole body inhalational exposure system which simulated “real‐world” environmental PM_2.5_ exposure. Mice continuous exposed to PM_2.5_ for 8 weeks demonstrated increased circulating serum CREA, urine α1‐MG, β2‐MG, Cys C, and RBP, and kidney injury score, indicating functional impairment and structure alteration in the kidney. KIM‐1, a sensitive kidney injury indicator, was found in the body fluid, renal cortical tissue and TECs, confirming the presence of kidney injury. In fact, a recent study in mice detected presence of PM_2.5_ in the kidney, an extrapulmonary organ,^[^ ^20^ ^]^ supporting kidney as the direct target organ of PM_2.5_. Because of the triggering role of kidney injury in kidney diseases, elucidating the underlying biological mechanism of kidney injury would contribute to exploring the protective strategies with early intervention.

Mitochondrial dysfunction is usually accompanied by oxidative stress, which is involved in most organismal chemical reactions and affects the degree of kidney disease.^[^ ^21^ ^]^ Free radical production, caused by the metal and organic components of PM_2.5_, leads to cell damage, which might be the initial source of organ injury.^[^ ^22^ ^]^ Tubular epithelial cells undergo subtle changes in response to initial injury and perform multiple biological functions in the kidneys.^[^ ^23^ ^]^ In the study, the balance of antioxidant enzymes and oxidase subunits became disturbed in the kidney and tubular epithelial cells, accompanied by enhanced ROS production and MMP reduction in vitro. Redox imbalance in the kidneys is known to trigger mitochondrial dysfunction. Thus, sustained oxidative stress followed by mitochondrial dysfunction may be a critical pathological event in kidney injury in response to PM_2.5_ exposure.

Mitochondrial dysfunction under oxidative stress can evoke autophagy to counteract excessive ROS production and maintain homeostasis.^[^ ^13^, ^24^ ^]^ Conversely, autophagy eliminates impaired intracellular mitochondria to maintain cellular homeostasis,^[^ ^25^ ^]^ which is essential for normal cell growth and proliferation. Generally, autophagy is regulated by Pro‐ and anti‐autophagy factors. mTOR and P62, which are critical negative regulatory autophagy factors, could be inhibited by oxidative stress.^[^ ^26^ ^]^ During the early stages of autophagosome formation, ATG5 and Beclin1 recruit autophagy‐related proteins and can directly activate autophagy signaling pathways.^[^ ^27^ ^]^ Meanwhile, LC3 and LC3I in the cytoplasm are transformed together into the membrane‐bound form of LC3II. The LC3II /LC3I ratio is usually used to evaluate autophagy. We observed that pro‐autophagy‐related molecules were upregulated, and anti‐autophagy‐related molecules (mTOR and P62) were downregulated in vivo and in vitro. The stimulation of pro‐autophagy molecules and the inhibition of anti‐autophagy factors suggest that autophagy contributes to PM_2.5_‐induced kidney injury.

Additionally, excessive ROS production can trigger pyroptosis, which is mediated by the NLRP3 inflammasome during the pathological process of renal diseases.^[^ ^28^ ^]^ The NLRP3 inflammasome comprises a group of multimeric protein complexes consisting of NLRP3, Caspase1 and ASC. Activated Caspase1 can cleave GSDMD to generate N‐terminal domain (N‐GSDMD) oligomers and form membrane pores leading to membrane rupture.^[^ ^29^ ^]^ Furthermore, activated Caspase1 also induces the maturation and secretion of various inflammatory cytokines, such as IL‐1β and IL‐18.^[^ ^30^ ^]^ In the present study, the levels of molecules in the pyroptosis pathway and secretory cytokines were upregulated in response to PM_2.5_ exposure under in vivo and in vitro conditions. These findings indicated that pyroptosis may be participated in the process of PM_2.5_‐induced kidney injury.

Given that intracellular ROS triggers autophagy and pyroptosis, we speculated that ROS elimination may be a strategy resisting PM_2.5_‐incurred mitochondrial dysfunction and kidney injury by inhibiting autophagy and pyroptosis. NAC was employed to experimentally interfere with excessive ROS in the renal TECs. As expected, NAC pre‐treatment inhibited excessive ROS production in PM_2.5_‐exposed HK‐2 cells. Accordingly, this intervention alleviated PM_2.5_‐induced injury to the TECs, attenuated pyroptosis, autophagy, mitochondrial damage and apoptosis. Furthermore, it was validated in animals which showed NAC treatment alleviated renal injury, redox imbalance and activation of autophagy and pyroptosis induced by PM_2.5_ exposure. Thus, these data suggest that ROS is the potential therapeutic target for alleviating nephrotoxicity induced by PM_2.5_ exposure and antioxidants or extracts derived from natural plants with antioxidant function may be the potential interventions as therapeutic strategy.

In summary, our findings indicate that PM_2.5_ exposure was associated with increased risk of kidney diseases and was a direct evoker of kidney injury. Although we did not elucidate the in‐depth mechanism by which PM_2.5_ induced oxidative stress, redox imbalance was identified as the key pathologic event in kidney injury which evoked mitochondrial damage and activated autophagy and pyroptosis (**Figure** **8**). Due to the ubiquity of PM_2.5_ in the air and the essential requisite of air for human beings, it is urgent to identify the nephrotoxicity of PM_2.5_. Meanwhile, further strategies for ROS intervention and efforts to improve air quality may ease the burden of kidney diseases. These findings shed new and direct light on PM_2.5_‐induced nephrotoxicity and potential therapeutic strategies.

## 4 Experimental Section

### Prospective Cohort Studies—Study Participants

First, this work performed a longitudinal analysis to assess the association between exposure to PM_2.5_ and the risk of incident kidney diseases based on the information of participants from the United Kingdom (UK) Biobank, which is a large‐scale epidemiological resource consisting of ≈0.5 million participants.^[^ ^31^ ^]^ Briefly, participants aged between 39 and 73 years attended one of the 22 assessment centers across the UK between 2006 and 2010, where they completed questionnaires, underwent physical measurements and provided biological samples. All participants provided informed consent, and the study was supported by the Northwest Multicenter Research Committee (11/NW/0382).

A flowchart of the study participants’ inclusion and exclusion criteria is shown in Figure S1, Supporting Information. Briefly, participants with prevalent kidney diseases or having renal replacement therapy at baseline (*n* = 13 033), and missing data on exposure to PM_2.5_ (*n* = 41 297) were excluded, and 448 085 participants remained in the analytical cohort.

### Prospective Cohort Studies—Collection of PM_2.5_ Concentrations

Estimates of ambient air pollutants were collected using the UK Biobank from 2005 to 2007 and 2010. For PM_2.5_, the annual concentration data were only available for 2010 and were therefore directly defined as their respective exposures at baseline. Mean annual concentrations of PM_2.5_ exposure were calculated using land‐use regression model, which is part of the European Study of Cohorts for Air Pollution Effects and were associated with the residential address provided by participants at the baseline visit.^[^ ^32^ ^]^ Model validation has been described elsewhere (http://www.escapeproject.eu/). Briefly, for PM_2.5_, leave‐one‐out cross‐validation where each site was ignored in turn, with the model's inclusion variables held constant, reveals good model performance (cross‐validation *R* ^2^ = 77%).

### Prospective Cohort Studies—Outcome Assessment

At baseline, self‐reported data was combined with the hospital inpatient records [using the International Classification of Diseases, Manual of the International Statistical Classification of Diseases, Injuries and Causes of Death (ICD‐9) or the International Statistical Classification of Diseases and Related Health Problems, Tenth Revision (ICD‐10)] to identify the prevalent cases of kidney diseases. Hospital inpatient records were directly linked to the Health Episode Statistics in England and Wales and the Scottish Morbidity Records in Scotland, which made it possible to accurately determine the date of first recording for each diagnosis.

After removing the prevalent kidney disease cases at baseline, this work identified the incident events of kidney diseases, including nephritis, hydronephrosis, kidney stones, acute renal failure, chronic kidney disease, and kidney cancer, during the subsequent follow‐up from the admission data using the ICD‐9 or ICD‐10 codes. The details of the codes used to identify kidney diseases in the present study are shown in Table S1, Supporting Information.

The follow‐up time was calculated from the baseline assessment or date of initial recruitment to the onset of kidney diseases or death, date of loss to follow‐up or date of the current end of follow‐up, whichever occurred first. Participants who were lost to follow‐up or died before kidney disease onset were censored at the time of the event.

### Prospective Cohort Studies—Covariates

Through a literature search, the potential confounders were identified as sociodemographic, behavioral and biomarker factors. Age was calculated using the date of birth and the date of the baseline assessment. Body mass index (BMI) was commonly used to evaluate the nutritional status or level of physical development of the human body and calculated from measured height and weight. Additionally, it was further defined as underweight (<18.5 kg m^−2^), normal weight (18.5 to <25 kg m^−2^), overweight (25 to <30 kg m^−2^) and obese (≥30 kg m^−2^). Education levels were defined as “college or university degree,” “professional education” and “other.” Participants with pre‐tax household income were divided into five levels. Smoking status was also an important variable, therefore the participants were divided into three further groups, including never smokers, former smokers, and current smokers. Drinking status was similarly classified. Multiple imputations were applied to impute missing covariate data, and the proportions of missing data were as follows: 7% for systolic blood pressure (SBP) and glycated haemoglobin (HbA1c); 1% for physical activity and pre‐tax household income; and <1% for ethnicity, education level, BMI, smoking status and drinking status. Additionally, we restricted the analysis to participants with complete covariate data for sensitivity analyses to examine the robustness of the results.

### Prospective Cohort Studies—Statistical Analysis

Continuous variables were presented as mean ± standard deviation (SD) and compared using Student's t‐test between low and high PM_2.5_ exposure groups, whereas categorical variables were presented as percentages and compared using the chi‐square test between groups. Cox proportional hazard models were used to estimate the associations between PM_2.5_ exposure and the risk of incident kidney diseases. Crude hazard ratios (HRs) with 95% confidence intervals (CIs) were calculated for Model 1 (crude model) and were adjusted for age, sex and ethnicity for Model 2 (basic model). Model 3 was further adjusted for educational level, average total household income before tax, physical activity, smoking status, drinking status, BMI, SBP and HbA1c levels (fully adjusted model). The proportional hazard assumption was evaluated by assessing the association between standardized Schoenfeld residuals and time. The concentration of PM_2.5_ exposure was regarded as a continuous variable and further categorized as low (<10 µg m^−3^) or high PM_2.5_ levels (≥10 µg m^−3^) based on the World Health Organization yearly air quality guideline values.^[^ ^33^ ^]^ Restricted cubic splines fitted with four knots were used to assess the potential non‐linear relationships between PM_2.5_ and the risk of incident kidney diseases.

All statistical analyses were performed using R version 4.2.1 unless otherwise noted. Statistical significance was set at two‐sided *p* < 0.05.

### In Vivo and In Vitro Studies—Animals

SPF‐grade 8‐week‐old male C57BL/6N mice were obtained from Beijing Vital River Laboratory Animal Technology Co., Ltd. (Beijing, China). The mouse experiment ensured that each group was assigned a minimum of 8 mice and mice with behavioral abnormalities or health problems were excluded, if any. Mice were housed under a 12‐h light/dark cycle in a temperature‐controlled room (22 ± 2 °C) with unrestricted access to abundant chow diet and water. This work was approved by the Institutional Animal Care and Use Committee of the Zhejiang Chinese Medical University.

### In Vivo and In Vitro Studies—PM_2.5_ Exposure Protocol and Sample Collection

This study included two independent animal experiments. In experiment 1, after a 2‐week acclimation, all mice were divided into two groups randomly, including filtered air (FA) and PM_2.5_ exposure groups (PM_2.5_). A whole‐body inhalation exposure system was used at Zhejiang Chinese Medical University in Hangzhou, China, where mice were exposed to FA or ambient PM_2.5_ for 12 h/d, 6 d/week, for 8 weeks. Detailed information on the system has been previously described.^[^ ^34^ ^]^ Briefly, the system was composed of two chambers. The PM_2.5_ chamber contained a cyclone connected to the ambient air to discharge the particulate matter >2.5 µm, and PM_2.5_ was collected ultimately. Contrastingly, FA chamber contained a particulate air filter to filter the air particulate matter.

In experiment 2, all mice were randomly divided into four groups: FA, FA+NAC, PM_2.5_ and PM_2.5_+NAC groups. All mice were exposed to FA or ambient PM_2.5_ in a whole‐body inhalation exposure system. In the FA+NAC and PM_2.5_+NAC groups, mice were administered with NAC (200 mg kg^−1^)^[^ ^35^ ^]^ by intraperitoneal injection daily for 8 weeks.

Mice were euthanized immediately after PM_2.5_ exposure for 8 weeks. Samples, including urine, serum and kidney tissues, were collected. Kidney samples were weighed to acquire the kidney coefficient, which is calculated by the ratio of kidney weight /body weight.

### In Vivo and In Vitro Studies—Biochemical Analysis

The levels of serum urea nitrogen (BUN), creatinine (CREA) and uric acid (UA) were measured by using a biochemical analyzer (HITACHI 3100 automatic biochemical analyzer). KIM‐1 proteins in serum and urine were determined using an ELISA kit (EMC018, Neobioscience Technology). The levels of urine α1‐microglobulin (α1‐MG), β2‐microglobulin (β2‐MG), cystatin C (Cys C) and retinol‐binding protein (RBP) were determined using ELISA kits (5045, 19 096, 10 030, 6498, MEIMIAN). 8‐Hydroxydesoxyguanosine(8‐OHdG) in serum was examined with an ELISA kit (ml002198, Mlbio). 4‐Hydroxynonenal (4‐HNE) in serum was determined using an ELISA kit (RXJ999214, Ruixin Biotech). The activity of Superoxide Dismutase (SOD), Heme oxygenase‐1 (HO‐1), Malondialdehyde (MDA) were determined using commercial assay kits (A001‐3‐2, H246‐1‐2, A003‐1‐2, Jiancheng Bioengineering Institute) according to the manufacturer’ s instructions.

### In Vivo and In Vitro Studies—Histology and Immunofluorescence Staining

Kidney tissues were immersed in 4% paraformaldehyde and then dehydration adequately before paraffin embedding. 4 µm kidney slides were used for Haematoxylin and Eosin (H&E) and Periodic Acid Schiff (PAS) staining. The degree of tissue damage was scored by the percentage of renal tubules with increased inflammatory cells, tubule dilatation and basement membrane thickening (0: no harm; 1: ≤25%; 2: 26%–50%; 3: 51%–75%; and 4: >75%) in a double‐blinded manner.

For immunofluorescence staining, de‐paraffinized tissue slides were blocked with 5% goat serum which was dissolved in phosphate‐buffered saline (PBS) containing 0.2% Triton X‐100 for 1 h at room temperature. The slides were covered with the KIM‐1 antibody (1:200; SAB3500252, Sigma) overnight at 4°C condition and washed 3 times with PBS before incubating in a corresponding secondary antibody (1:500; AS053, Abclonal) 1 h at room temperature. After applying DAPI staining solution (P0131, Beyotime), images were captured using fluorescence microscopy.

### In Vivo and In Vitro Studies—Cell Culture and PM_2.5_ Treatment

The human kidney proximal tubular epithelial cell line (HK‐2, CRL‐2190, ATCC) were cultured in DMEM/F12 medium (C11330500BT, Hyclone), and contained with 10% fetal bovine serum (FBS; F8318, Sigma) and 1% penicillin/streptomycin solution (P/S; V900929, Sigma). The cells were serum‐starved overnight (12 h) after seeding into well plates of different sizes at 37 °C in a 5% CO_2_ atmosphere. PM_2.5_ (SRM1648a, NIST) was suspended and dissolved to the appropriate concentrations in the medium, and the HK‐2 cells were treated with different concentrations (0, 25, 50, and 100 µg mL^−1^) of PM_2.5_ for 24 h.

### In Vivo and In Vitro Studies—Quantitative Real‐Time Polymerase Chain Reaction

Total RNA was separated from the kidney tissues and HK‐2 cells using RNAiso Plus kit (9108, TaKaRa). cDNA was synthesized using the PrimeScript RT Mix (6210, TaKaRa). Using SYBR Green Mix (A25742, Themo) in a QuantStudio 7 Flex (Themo) to complete qRT‐PCR process. mRNA levels were performed by the 2^−ΔΔCt^ method and normalized to the β‐actin expression. The primer sequences are listed in Table S4, Supporting Information.

### In Vivo and In Vitro Studies—Western Blot Analysis

Kidney tissue and HK‐2 cell samples were homogenized in RIPA lysis buffer (AR0102, Boster), then the concentrations of the obtained proteins were determined using a bicinchoninic acid Protein Assay Kit (P0010S, Beyotime). Samples were separated using SDS‐PAGE and transferred to polyvinylidene difluoride (PVDF) membranes. The PVDF membrane were blocked 1 h with 5% bovine serum albumin (BSA, ST023, Beyotime) at room temperature. Primary antibodies included NLRP3 antibody (15 101), ASC antibody (67 824), GSDMD antibody (39 754), caspase‐1 antibody (83 383), BAX antibody (SAB4502546), Bcl‐2 antibody (SAB4500003), mTOR antibody (2983S), LC3B antibody (12 741), P62 antibody (5114S), Beclin1 antibody (3495), ATG5 antibody (12 994), GAPDH antibody (5174S) and β‐Tubulin antibody (2128S). These antibodies were obtained from Cell Signaling Technology and at a dilution of 1:1000. Then the secondary antibody was coated on the PVDF membranes, HRP Goat Anti‐Rabbit IgG (A0208) and HRP Goat Anti‐Mouse IgG (A0216) were obtained from Beyotime and at a dilution of 1:5000. Imaging was then performed in a ChemiDoc imaging system (Bio‐Rad) using enhanced chemiluminescence (ECL) reagents. The captured images were analyzed using ImageJ.

### In Vivo and In Vitro Studies—Annexin V and Propidium Iodide Staining Assay

Cell apoptosis was evaluated using FITC Annexin V with PI kit (C1062S, Beyotime). Shortly, HK‐2 cells treated with different PM_2.5_ concentrations were washed with PBS and resuspended in 195 µL binding buffer, which included 5 µL Annexin‐V and 10 µL PI staining solution. After adding another appropriate binding buffer, the cells were analyzed using flow cytometry (DxFLEX, Beckman). The apoptotic cells ratio was analyzed using FlowJo.

### In Vivo and In Vitro Studies—Determining the ROS Levels and MMP (ΔΨm)

2,7‐dichlorofluorescein diacetate (DCFH‐DA) probes (S0033S, Beyotime) were used to evaluate intracellular ROS levels in HK‐2 cells. Briefly, treated cells were transferred to PBS contained with 10 µM DCFH‐DA for 20 min at 37 °C without light. Fluorescence value in all sample was calculated using flow cytometry (DxFLEX; Beckman). The results were analyzed using FlowJo.

ΔΨm of PM_2.5_‐treated HK‐2 cells were measured using a mitochondrial membrane potential assay kit (C2003S, Beyotime), of which the main active reagent is fluorescent dye 5,5′,6,6′‐tetrachloro‐1,1′,3,3′‐tetraethylbenzimidazol‐carbocyanine iodide (JC‐1). HK‐2 cells were incubated with JC‐1 dye for 20 min in the dark. HK‐2 cells were incubated with JC‐1 dye for 20 min in the dark. The green fluorescence represents monomeric form, indicating depolarized/low ΔΨm, and the red fluorescence represent aggregated form, indicating polarized/normal ΔΨm. HK‐2 cells loaded with JC‐1 probes were examined under a fluorescence microscope (Leica, 20 × 10). The red/green fluorescence intensity ratio represents ΔΨm using Image‐Pro Plus 6.0. Additionally, the MitoTracker Green (C1048, Beyotime) and MitoTracker Red CMXRos (C1049B, Beyotime) were also used to measure MMP of HK‐2 cells by flow cytometry. Data were analyzed using FlowJo.

### In Vivo and In Vitro Studies—Determination of Mitochondrial Oxygen Consumption Rate

20 mg of fresh kidney tissue was taken and added to the respiratory solution and homogenized thoroughly, the homogenate was then added to Oroboros O2k and pyruvate, glutamate, malate, the substrates of complex I were added to obtain the leak of complex I. After the respiratory values were stabilized, ADP, succinate, CCCP, rotenone, Antimycin A were added to obtain the maximum oxidative phosphorylation values and maximum electron transfer capacity values of complexes I and II, respectively.

### In Vivo and In Vitro Studies—Statistical Analysis

The hazard ratios (HR) and 95% confidence intervals (CI) of the associations between exposure to PM_2.5_ and the risk of incident kidney diseases were estimated using Cox proportional hazard models. Crude HRs with 95% CIs were calculated in Model 1 (crude model) and adjusted for age, sex and ethnicity in Model 2 (basic model). Model 3 was further adjusted for educational level, average total household income before tax, physical activity, smoking status, drinking status, BMI, SBP and HbA1c levels (fully adjusted model). The association between standardized Schoenfeld residuals and time was assessed to evaluate the proportional hazard assumption. The concentration of PM_2.5_ exposure was regarded as a continuous variable and further categorized as low (<10 µg m^−3^) and high PM_2.5_ pollution (≥10 µg m^−3^) based on the World Health Organization yearly air quality guideline values.^[^ ^33^ ^]^ Restricted cubic splines fitted with four knots were used to assess the potential non‐linear relationships between PM_2.5_ and the risk of incident kidney diseases.

All data from in vivo and in vitro experiments were presented as mean ± standard error of mean (SEM). Student's *t*‐test was used to compare data between two groups and one‐way ANOVA was used to compare data between multiple groups The GraphPad Prism 9.3 was used for statistical analyses, and *p* < 0.05 generally considered statistically significant.

## Conflict of Interest

The authors declare no conflict of interest.

## Author Contributions

T.H. and Y.J. contributed equally to this work. C.L. and Y.M. designed this study. T.H., R.H., and J.Z. performed animal experiments. Y.J. performed the population data analysis. T.H., S.L., and W.F. performed the histology and imaging analysis. T.H. and J.Z. performed cell culture and in vitro experiments. R.C., L.Z., R.L., L.Q., W.G., Y.W., L.Z., X.Z., and Q.S. provided technical or material support and consultation. T.H. and Y.J. wrote the original draft. C.L. and Y.M. reviewed and revised the manuscript. C.L. approved the final version.

## Data and Materials Availability

All data needed to evaluate the conclusions in the paper are present in the paper and/or the Supplementary Materials. Additional data related to this paper may be requested from the authors. The individual‐level phenotype data requires permission from the UK Biobank and the code is available upon request.

## Supporting information



Supporting Information

## Data Availability

The data that support the findings of this study are available from the corresponding author upon reasonable request.

## References

[1] a) F. Liu , G. Chen , W. Huo , C. Wang , S. Liu , N. Li , S. Mao , Y. Hou , Y. Lu , H. Xiang , Environ. Pollut. 2019, 252, 1235; 31252121 10.1016/j.envpol.2019.06.033

[2] a) S. Y. Lin , S. W. Ju , C. L. Lin , W. H. Hsu , C. C. Lin , I. W. Ting , C. H. Kao , Environ. Pollut. 2020, 261, 114154; 32088432 10.1016/j.envpol.2020.114154

[3] P. Romagnani , G. Remuzzi , R. Glassock , A. Levin , K. J. Jager , M. Tonelli , Z. Massy , C. Wanner , H. J. Anders , Nat. Rev. Dis. Primers 2017, 3, 17088. 29168475 10.1038/nrdp.2017.88

[4] N. Jourde‐Chiche , F. Fakhouris , L. Dou , J. Bellien , S. Burtey , M. Frimat , P. A. Jarrot , G. Kaplanski , M. Le Quintrec , V. Pernin , C. Rigothier , M. Sallée , V. Fremeaux‐Bacchi , D. Guerrot , L. T. Roumenina , Nat. Rev. Nephrol. 2019, 15, 87. 30607032 10.1038/s41581-018-0098-z

[5] L. Yao , X. Liang , Y. Qiao , B. Chen , P. Wang , Z. Liu , Metabolism. 2022, 131, 155195. 35358497 10.1016/j.metabol.2022.155195

[6] J. Bhandari , P. K. Thada , H. Arif , StatPearls Publishing Copyright © 2022, StatPearls Publishing LLC, Treasure Island (FL) 2022.

[7] Y. Liu , X. He , J. Liu , L. Zhang , A. Xiong , J. Wang , S. Liu , M. Jiang , L. Luo , Y. Xiong , G. Li , Ecotoxicol. Environ. Saf. 2022, 244, 114039. 36049333 10.1016/j.ecoenv.2022.114039

[8] a) Y. G. Kim , S. M. Kim , K. P. Kim , S. H. Lee , J. Y. Moon , Cells. 2019, 8, 1389;31694192

[9] D. Cui , S. Liu , M. Tang , Y. Lu , M. Zhao , R. Mao , C. Wang , Y. Yuan , L. Li , Y. Chen , J. Cheng , Y. Lu , J. Liu , Phytomedicine. 2020, 66, 153111. 31790902 10.1016/j.phymed.2019.153111

[10] a) A. K. Aranda‐Rivera , A. Cruz‐Gregorio , O. E. Aparicio‐Trejo , J. Pedraza‐Chaverri , Biomolecules. 2021, 11, 1144; 34439810 10.3390/biom11081144 PMC8391472

[11] S. van der Rijt , J. C. Leemans , S. Florquin , R. H. Houtkooper , A. Tammaro , Nat. Rev. Nephrol. 2022, 18, 588. 35798902 10.1038/s41581-022-00592-x

[12] O. Van Aken , F. Van Breusegem , Trends Plant Sci. 2015, 20, 754. 26442680 10.1016/j.tplants.2015.08.002

[13] Y. N. Qiu , G. H. Wang , F. Zhou , J. J. Hao , L. Tian , L. F. Guan , X. K. Geng , Y. C. Ding , H. W. Wu , K. Z. Zhang , Ecotoxicol. Environ. Saf. 2019, 167, 178. 30336408 10.1016/j.ecoenv.2018.08.050

[14] a) H. Zhang , R. Xu , Z. Wang , Oxid. Med. Cell Longev. 2021, 2021, 6114132; 34712385 10.1155/2021/6114132 PMC8548138

[15] R. Hu , L. Zhang , L. Qin , H. Ding , R. Li , W. Gu , R. Chen , Y. Zhang , S. Rajagoplan , K. Zhang , Q. Sun , C. Liu , Environ. Pollut. 2023, 324, 121347. 36858098 10.1016/j.envpol.2023.121347

[16] E. Latoch , K. Konończuk , K. Muszyńska‐Rosłan , K. Taranta‐Janusz , A. Wasilewska , E. Szymczak , J. Trochim , M. Krawczuk‐Rybak , J Clin Med. 2021, 10, 399. 33494327 10.3390/jcm10030399 PMC7866176

[17] S. Copur , D. Ucku , M. Kanbay , Clin. Kidney J. 2022, 15, 1800. 36158144 10.1093/ckj/sfac101 PMC9494525

[18] L. M. A. Barnett , B. S. Cummings , Toxicol. Sci. 2018, 164, 379. 29939355 10.1093/toxsci/kfy159

[19] J. A. Kellum , P. Romagnani , G. Ashuntantang , C. Ronco , A. Zarbock , H. J. Anders , Nat. Rev. Dis. Primers. 2021, 7, 52. 34267223 10.1038/s41572-021-00284-z

[20] D. Li , Y. Li , G. Li , Y. Zhang , J. Li , H. Chen , Proc. Natl. Acad. Sci. U S A. 2019, 116, 2488. 30692265 10.1073/pnas.1818134116 PMC6377456

[21] H. J. Ho , H. Shirakawa , Cells. 2022, 12, 88. 36611880 10.3390/cells12010088 PMC9818928

[22] K. Liu , S. Hua , L. Song , Oxid. Med. Cell Longev. 2022, 2022, 3618806. 35419163 10.1155/2022/3618806 PMC9001082

[23] J. H. Ix , M. G. Shlipak , Am. J. Kidney Dis. 2021, 78, 719. 34051308 10.1053/j.ajkd.2021.03.026 PMC8545710

[24] L. J. Su , J. H. Zhang , H. Gomez , R. Murugan , X. Hong , D. Xu , F. Jiang , Z. Y. Peng , Oxid. Med. Cell Longev. 2019, 2019, 5080843. 31737171 10.1155/2019/5080843 PMC6815535

[25] a) D. D. Xu , L. L. Du , Cells. 2022, 11, 1086;35406650

[26] Y. Wang , M. Tang , Sci. Total Environ. 2020, 710, 136397. 32050373 10.1016/j.scitotenv.2019.136397

[27] B. Shi , M. Ma , Y. Zheng , Y. Pan , X. Lin , J. Cell. Physiol. 2019, 234, 12562. 30618070 10.1002/jcp.28125 PMC13484135

[28] Q. Lin , S. Li , N. Jiang , X. Shao , M. Zhang , H. Jin , Z. Zhang , J. Shen , Y. Zhou , W. Zhou , L. Gu , R. Lu , Z. Ni , Redox Biol. 2019, 26, 101254. 31229841 10.1016/j.redox.2019.101254 PMC6597739

[29] N. Kelley , D. Jeltema , Y. Duan , Y. He , Int. J. Mol. Sci. 2019, 20, 3328. 31284572 10.3390/ijms20133328 PMC6651423

[30] N. Van Opdenbosch , M. Lamkanfi , Immunity. 2019, 50, 1352. 31216460 10.1016/j.immuni.2019.05.020 PMC6611727

[31] C. Sudlow , J. Gallacher , N. Allen , V. Beral , P. Burton , J. Danesh , P. Downey , P. Elliott , J. Green , M. Landray , B. Liu , P. Matthews , G. Ong , J. Pell , A. Silman , A. Young , T. Sprosen , T. Peakman , R. Collins , PLoS Med. 2015, 12, e1001779. 25826379 10.1371/journal.pmed.1001779 PMC4380465

[32] M. Eeftens , R. Beelen , K. de Hoogh , T. Bellander , G. Cesaroni , M. Cirach , C. Declercq , A. Dėdelė , E. Dons , A. de Nazelle , K. Dimakopoulou , K. Eriksen , G. Falq , P. Fischer , C. Galassi , R. Gražulevičienė , J. Heinrich , B. Hoffmann , M. Jerrett , D. Keidel , M. Korek , T. Lanki , S. Lindley , C. Madsen , A. Mölter , G. Nádor , M. Nieuwenhuijsen , M. Nonnemacher , X. Pedeli , O. Raaschou‐Nielsen , et al., Environ. Sci. Technol. 2012, 46, 11195. 22963366 10.1021/es301948k

[33] A. Goshua , C. A. Akdis , K. C. Nadeau , Allergy. 2022, 77, 1955. 35060140 10.1111/all.15224 PMC12052406

[34] R. Li , Q. Sun , S. M. Lam , R. Chen , J. Zhu , W. Gu , L. Zhang , H. Tian , K. Zhang , L. C. Chen , Q. Sun , G. Shui , C. Liu , Part. Fibre Toxicol. 2020, 17, 14. 32321544 10.1186/s12989-020-00343-5 PMC7178763

[35] K. L. Guss , S. Pavanni , B. Prati , L. Dazzi , J. P. de Oliveira , B. V. Nogueira , T. M. C. Pereira , M. Fronza , D. C. Endringer , R. Scherer , Ultrason. Sonochem. 2017, 37, 368. 28427645 10.1016/j.ultsonch.2017.01.035

