# Supplementary material for: Kidney Injury Evoked by Fine Particulate Matter: Risk Factor, Causation, Mechanism and Intervention Study
Source: Adv Sci (Weinh). 2024 Sep 24;11(43):2403222. doi: 10.1002/advs.202403222 (PMC11578332; doi:10.1002/advs.202403222)
Supplement: Supplementary file 1 — Supporting Information [file ADVS-11-2403222-s001.docx]

Supplemental information includes six figures and four tables.

**Figure S1**

Flowchart of participants inclusion and exclusion based on the UK Biobank.


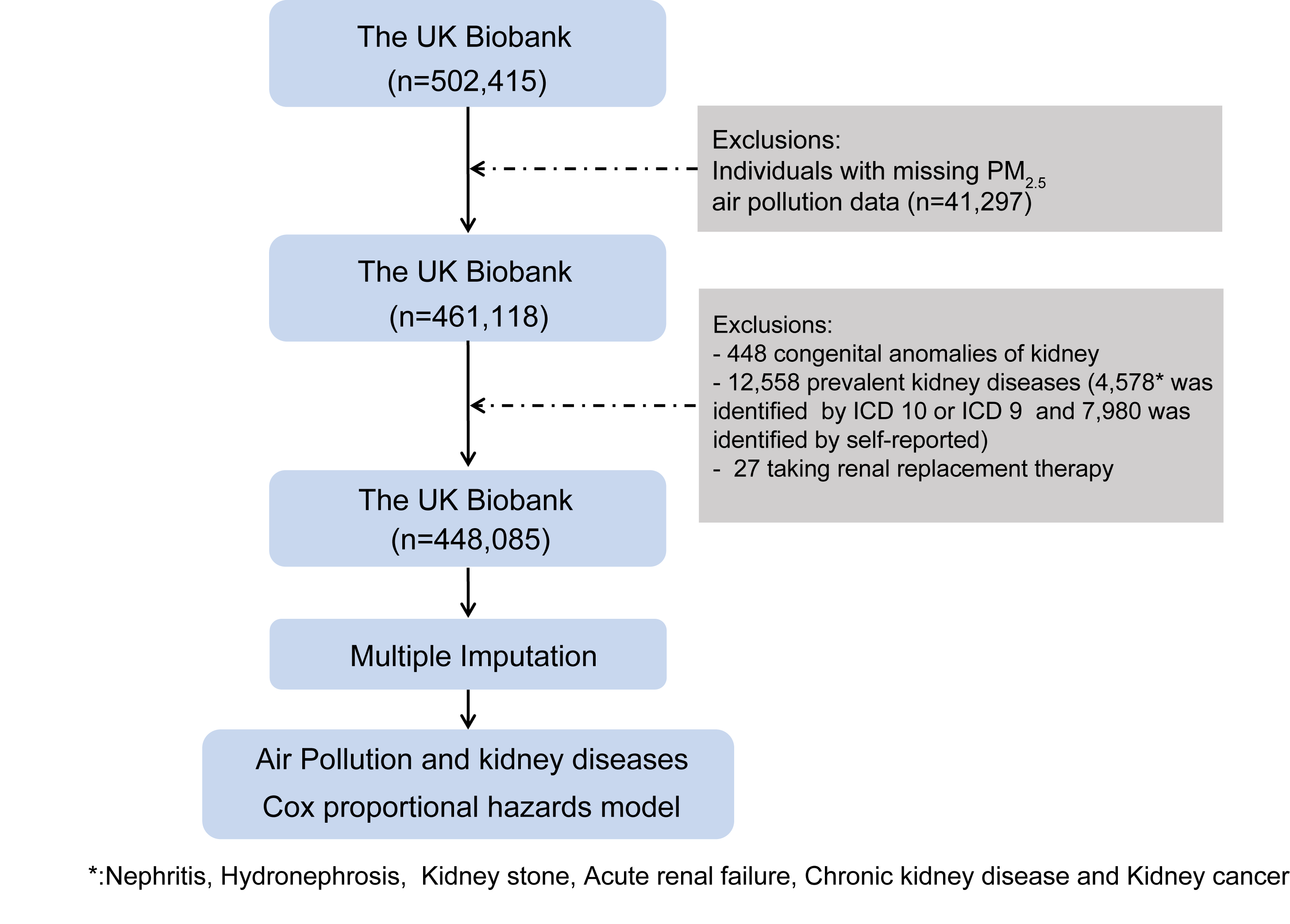


**Figure S2**

Dose-response relationships of exposure to PM_2.5_ with the risk of kidney diseases.


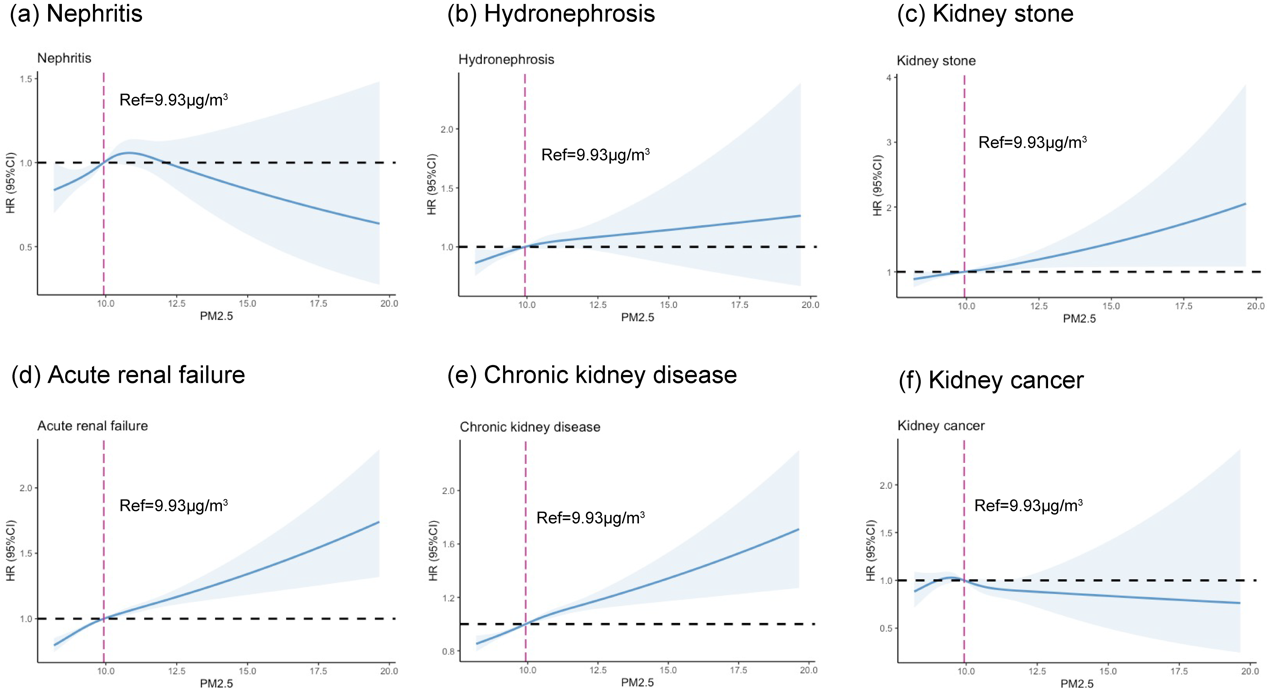


**Figure S3**

**
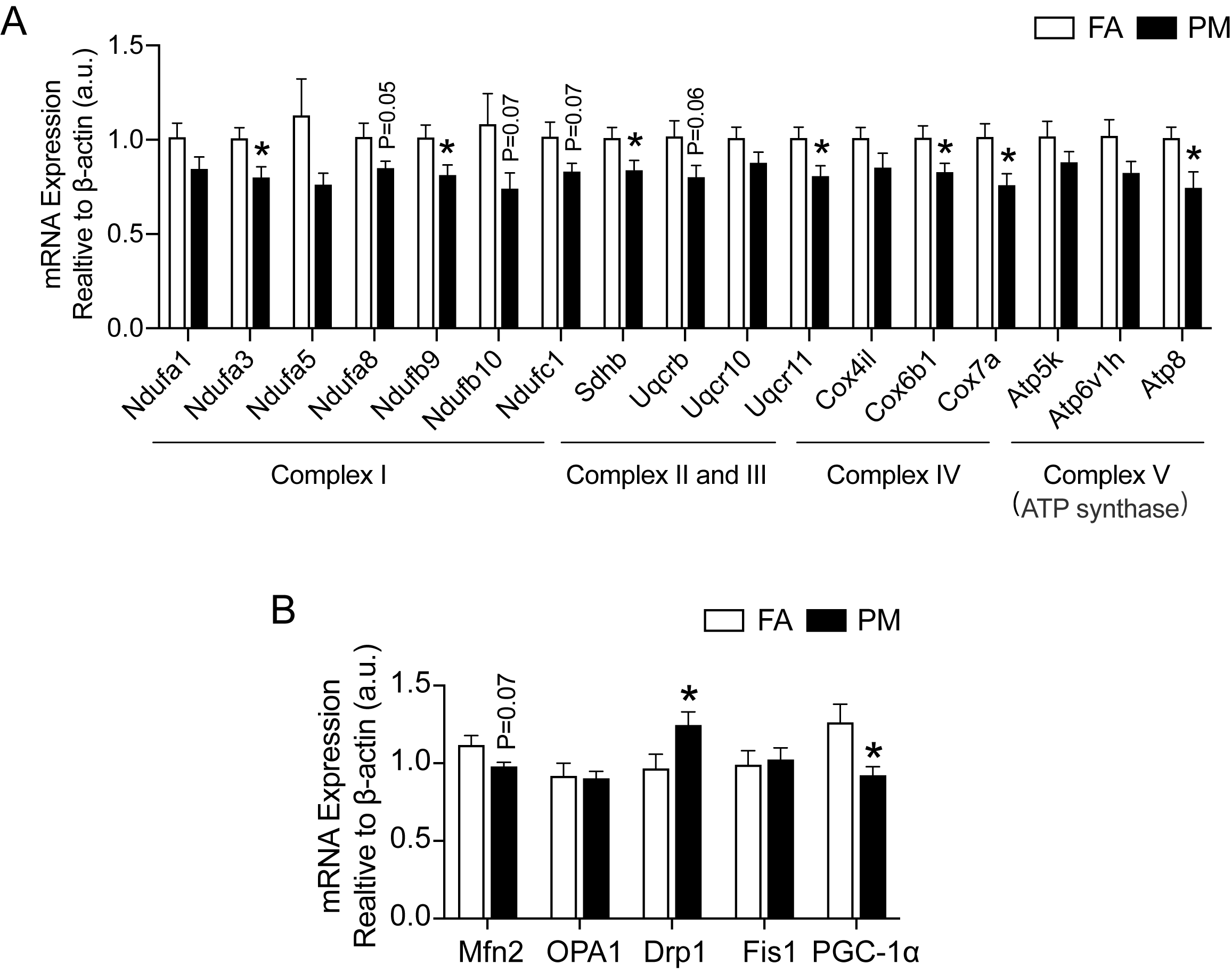
**

Figure S3. Effects of PM_2.5_ exposure on mitochondrial function and morphology in the kidney. (A) The gene expression of CI, CII, CIII, CIV and CV in HK-2 cells was quantitated using qRT-PCR. (B) The gene expression of Mfn2, OPA1, Drp1, Fis1 and PGC-1α in HK-2 cells was quantitated using qRT-PCR. (n=8) (**p* < 0.05 vs. FA group.)

**Figure S4**

**
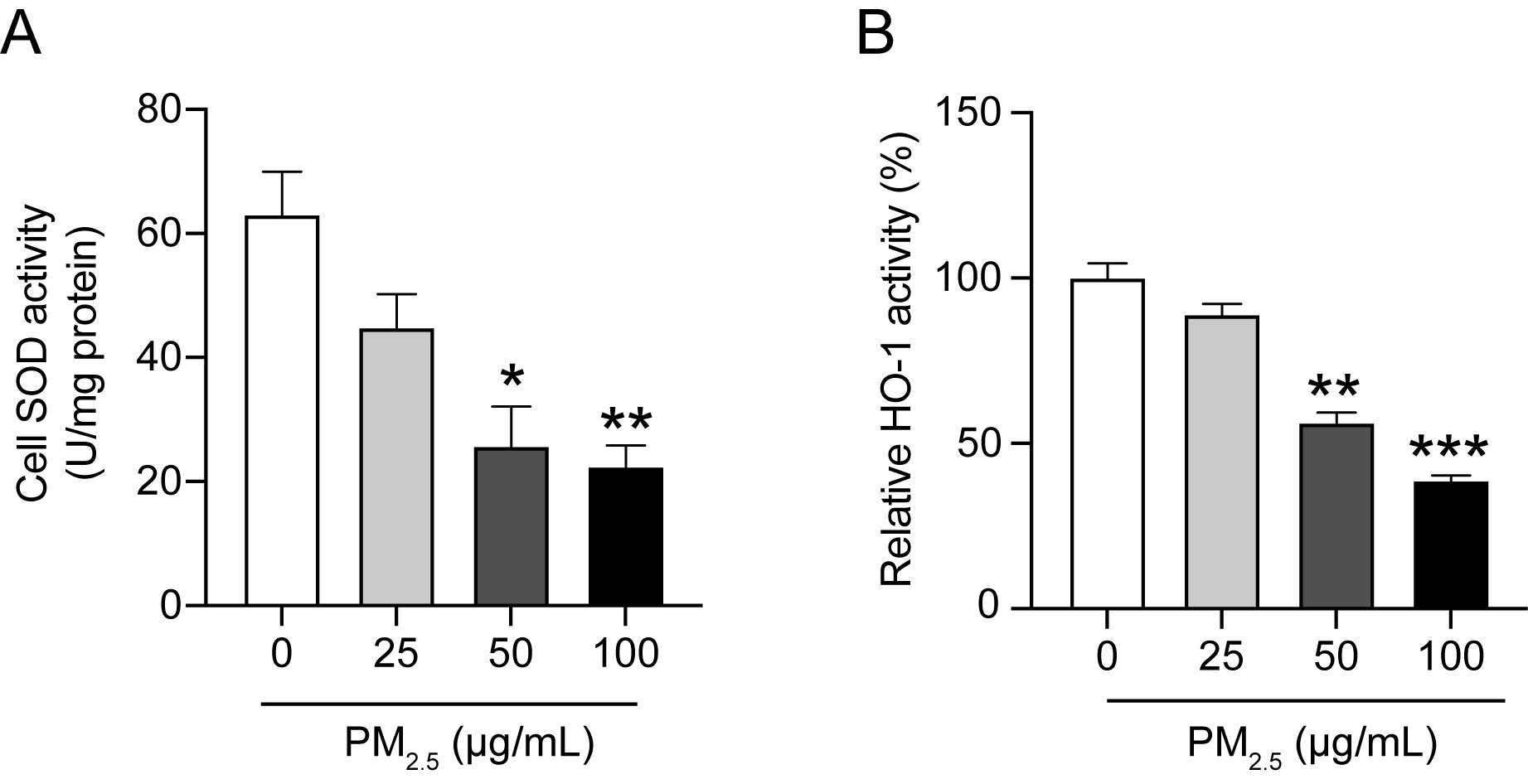
**

Figure S4. Effects of PM_2.5_ exposure on SOD and HO-1 activities in HK-2 cells. (A) ELISA assay for SOD activities in HK-2 cells after different treatments. (B) ELISA assay for relative HO-1 expression in HK-2 cells. (n=3) (**p* < 0.05, ***p* < 0.01 and ****p* < 0.001 vs. 0 μg/mL group.)

**Figure S5**


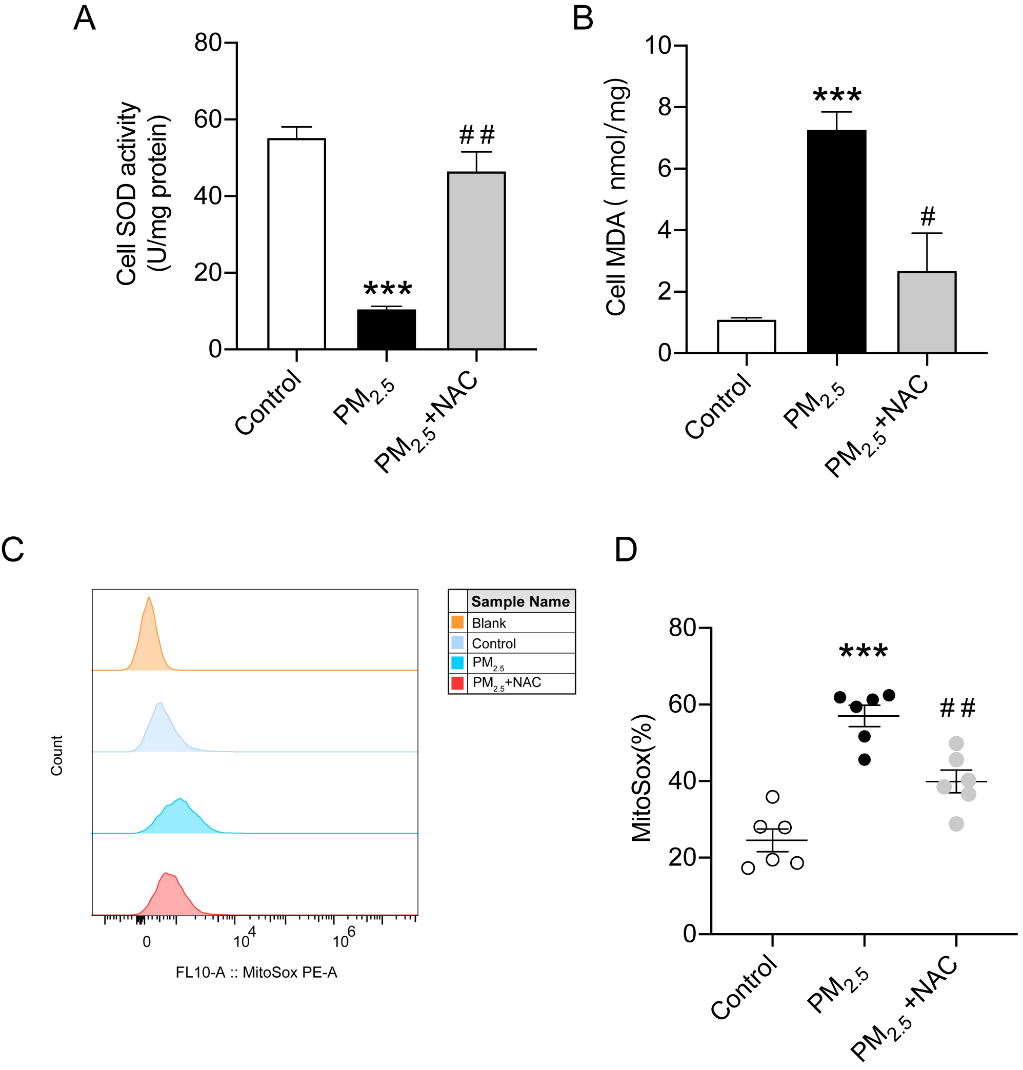


Figure S5. Effects of NAC treatment on oxidative stress in HK-2 cells exposed to PM_2.5_. (A) ELISA assay for SOD activities in HK-2 cells. (B) ELISA assay for MDA content in HK-2 cells. (C) The MitoSox levels of HK-2 cells were determined by flow cytometry. (D) MitoSox ratio. (n=6) (****p* < 0.001 vs. Control group; ^#^*p* < 0.05, ^# #^*p* < 0.01 vs. PM_2.5_ group.)

**Figure S6**

**
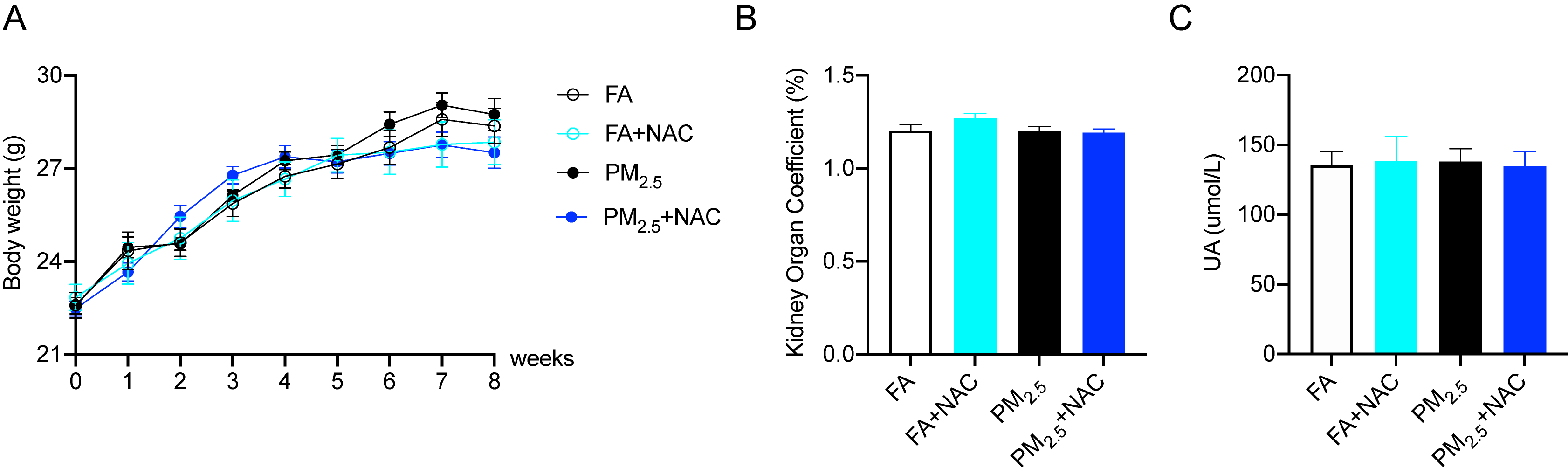
**

Figure S6. Effects of NAC treatment on body weight, kidney organ coefficient and UA in mice exposed to PM_2.5_. (A) Body weight (B) Kidney Organ Coefficient. (C) Serum level of UA in mice. (n=8)

**Table S1**

Source and corresponding code to identify cases with kidney diseases in the UK Biobank.

| Source | UK Biobank data fields | Corresponding code |
| --- | --- | --- |
| Nephritis | 41720: “Diagnoses - ICD10”  41271: “Diagnoses - ICD9” | ICD-9: 582, 5829, 583, 5830, 5831  ICD-10: N00, N01, N03, N06.2, N06.3, N06.7, N07.2, N07.3, N07.5, N07.7, N10, N11, N12 |
| Hydronephrosis |  | ICD-9: 591, 5919  ICD-10: N13.0, N13.1, N13.2, N13.3 |
| Kidney stone |  | ICD-9: 592, 5920, 5921, 5929  ICD-10: N20.0, N20.2 |
| Acute renal failure |  | ICD-9: 584, 5845, 5849  ICD-10: N17 |
| Chronic kidney disease |  | ICD-9: 585, 5859  ICD-10: N18 |
| Kidney cancer |  | ICD-9: 189，1890  ICD-10: C64 |
| Congenital anomalies of kidney |  | ICD-9: 7530, 7531, 7532, 7533  ICD-10: Q60, Q61, Q62, Q63 |
| Kidney replacement therapy |  | ICD-9: /  ICD-10: E85.3, N16.5, Q60.1, T82.4, T86.1, Y60.2, Y61.2, Y62.2, Y84.1, Z49.0, Z49.1, Z49.2, Z94.0, Z99.2 |
| Self-report kidney diseases | 20002: “non-cancer illness code, self-reported” | 1192, 1193, 1194, 1196, 1197, 1405, 1427, 1519, 1520, 1607, 1608, 1609 |

**Table S2**

Distribution of population characteristics in the not-imputed dataset.

| Characteristic | Low PM_2.5_ pollution^§^（n=213322） | High PM_2.5_ pollution^§^（n=186649） |
| --- | --- | --- |
| Sex, % |  |  |
| Male | 45.4(n=96878) | 45.8(n=85505) |
| Female | 54.6(n=116444) | 54.2(n=101144) |
| Age, y, mean (SD) | 56.99(7.96) | 55.97(8.21) |
| Age, % |  |  |
| <60 years | 54.3(n=115814) | 59.4(n=110897) |
| ≥60 years | 45.7(n=97508) | 40.6(n=75752) |
| Race/ethnicity, n. (%) |  |  |
| White | 96.6(n=206170) | 92.7(n=172967) |
| Others | 3.4(n=7152) | 7.3(n=13682) |
| BMI, kg/m^2^, mean (SD) | 27.22(4.56) | 27.54(4.91) |
| BMI, % |  |  |
| Underweight (<18.5 kg/m^2^) | 0.5(n=962) | 0.6(n=1084) |
| Normal (18.5-24.9 kg/m^2^) | 33.4(n=71302) | 32.1(n=59977) |
| Overweight (25-29.9 kg/m^2^) | 43.5(n=92715) | 41.7(n=77779) |
| Obesity (≥30 kg/m^2^) | 22.7(n=48343) | 25.6(n=47809) |
| Education level, % |  |  |
| College or university degree | 38.6(n=82378) | 39.3(n=73423) |
| Professional education | 55.0(n=117229) | 54.7(n=102092) |
| Other | 6.4(n=13715) | 6.0(n=11134) |
| Household income, £, % |  |  |
| Less than 18,000 | 19.3(n=41238) | 26.2(n=48928) |
| 18,000 to 31,000 | 25.3(n=53977) | 25.7(n=48004) |
| 31,000 to 52,000 | 27.1(n=57754) | 25.0(n=46744) |
| 52,000 to 100,000 | 22.3(n=47597) | 18.2(n=34028) |
| Greater than 100,000 | 6.0(n=12756) | 4.8(n=8945) |
| Smoking status, % |  |  |
| Never | 56.8(n=121228) | 52.6(n=98137) |
| Previous | 34.8(n=74151) | 35.0(n=65280) |
| Current | 8.4(n=17943) | 12.4(n=23232) |
| Drinking status, % |  |  |
| Never | 3.7(n=7787) | 5.0(n=9324) |
| Previous | 3.0(n=6297) | 4.0(n=7496) |
| Current | 93.4(n=199238) | 91.0(n=169829) |
| Physical activity, % |  |  |
| Yes | 26.5(n=56594) | 29.2(n=54535) |
| No | 73.5(n=156728) | 70.8(n=132114) |
| Systolic blood pressure (SBP;mmHg)* | 140.30(n=19.65) | 139.00(19.61) |
| Glycated hemoglobin (HbA1c; mmol/mol) * | 35.93(n=6.41) | 36.24(6.94) |
| Nephritis cases, % | 0.5(n=1010) | 0.6(n=1050) |
| Hydronephrosis cases, % | 0.8(n=1747) | 0.9(n=1637) |
| Kidney stone cases, % | 0.6(n=1381) | 0.7(n=1377) |
| Acute renal failure cases, % | 3.4(n=7219) | 4.0(n=7424) |
| Chronic kidney disease, % | 3.2(n=6729) | 3.6(n=6771) |
| Kidney cancer, % | 0.3(n=709) | 0.3(n=611) |

Definition of abbreviations: PM_2.5_ = particulate matter <2.5µm

§: Defined by WHO guideline value of PM_2.5_: low (<10 μg/m^3^) and high (>10 μg/m^3^).

*: Mean and standard deviation.

**Table S3**

Sensitivity analyses restricted to participants with complete covariates.

| Kidney diseases | Incidence  /Person-years | Model 1 | | Model 2^*^ | | Model 3^†^ | |
| --- | --- | --- | --- | --- | --- | --- | --- |
|  |  | HR (95%CI) | P Value | HR (95%CI) | P Value | HR (95%CI) | P Value |
| **Nephritis** | | | | | | | |
| PM_2.5_ pollution(μg/m^3^) | 2060/4696294 | 1.08(1.04-1.12) | 1.44×10^-4^ | 1.10(1.05-1.14) | 6.08×10^-6^ | 1.04(1.00-1.09) | 0.05017 |
| PM_2.5_ pollution (per IQR) | 2060/4696294 | 1.1(1.05-1.16) | 1.44×10^-4^ | 1.12(1.07-1.18) | 6.08×10^-6^ | 1.05(1.00-1.11) | 0.05017 |
| Low PM_2.5_ pollution^§^ | 1010/2504284 | Reference | Reference | Reference | Reference | Reference | Reference |
| High PM_2.5_ pollution^§^ | 1050/2192010 | 1.19(1.09-1.29) | 9.8×10^-5^ | 1.22(1.12-1.33) | 6.10×10^-6^ | 1.12(1.02-1.22) | 0.01451 |
| **Hydronephrosis** | | | | | | | |
| PM_2.5_ pollution(μg/m^3^) | 3384/4697546 | 1.04(1.01-1.08) | 0.00639 | 1.07(1.04-1.10) | 2.04×10^-5^ | 1.04(1.01-1.08) | 0.00714 |
| PM_2.5_ pollution (per IQR) | 3384/4697546 | 1.06(1.02-1.1) | 0.00639 | 1.09(1.05-1.13) | 2.04×10^-5^ | 1.06(1.02-1.10) | 0.00714 |
| Low PM_2.5_ pollution^§^ | 1747/2504874 | Reference | Reference | Reference | Reference | Reference | Reference |
| High PM_2.5_ pollution^§^ | 1637/2192671 | 1.07(1-1.14) | 0.0532 | 1.12(1.05-1.20) | 0.00104 | 1.07(1.00-1.15) | 0.04673 |
| **Kidney stone** | | | | | | | |
| PM_2.5_ pollution(μg/m^3^) | 2758/4695676 | 1.1(1.07-1.14) | 1.46×10^-8^ | 1.11(1.07-1.15) | 4.71×10^-9^ | 1.07(1.04-1.11) | 5.43×10^-5^ |
| PM_2.5_ pollution (per IQR) | 2758/4695676 | 1.13(1.09-1.18) | 1.46×10^-8^ | 1.14(1.09-1.19) | 4.71×10^-9^ | 1.10(1.05-1.14) | 5.43×10^-5^ |
| Low PM_2.5_ pollution^§^ | 1381/2504108 | Reference | Reference | Reference | Reference | Reference | Reference |
| High PM_2.5_ pollution^§^ | 1377/2191568 | 1.14(1.06-1.23) | 6.28×10^-4^ | 1.15(1.07-1.24) | 2.72×10^-4^ | 1.09(1.01-1.17) | 0.03136 |
| **Acute renal failure** | | | | | | | |
| PM_2.5_ pollution(μg/m^3^) | 14643/4676221 | 1.11(1.09-1.12) | <2×10^-16^ | 1.15(1.13-1.16) | <2×10^-16^ | 1.08(1.06-1.09) | <2×10^-16^ |
| PM_2.5_ pollution (per IQR) | 14643/4676221 | 1.14(1.11-1.16) | <2×10^-16^ | 1.19(1.17-1.21) | <2×10^-16^ | 1.10(1.08-1.12) | <2×10^-16^ |
| Low PM_2.5_ pollution^§^ | 7219/2494734 | Reference | Reference | Reference | Reference | Reference | Reference |
| High PM_2.5_ pollution^§^ | 7424/2181486 | 1.17(1.14-1.21) | <2×10^-16^ | 1.26(1.22-1.30) | <2×10^-16^ | 1.12(1.08-1.16) | 1.01×10^-11^ |
| **Chronic kidney disease** | | | | | | | |
| PM_2.5_ pollution(μg/m^3^) | 13500/4691741 | 1.08(1.07-1.1) | <2×10^-16^ | 1.13(1.11-1.15) | <2×10^-16^ | 1.07(1.06-1.09) | <2×10^-16^ |
| PM_2.5_ pollution (per IQR) | 13500/4691741 | 1.11(1.08-1.13) | <2×10^-16^ | 1.17(1.14-1.19) | <2×10^-16^ | 1.09(1.07-1.11) | <2×10^-16^ |
| Low PM_2.5_ pollution^§^ | 6729/2503222 | Reference | Reference | Reference | Reference | Reference | Reference |
| High PM_2.5_ pollution^§^ | 6771/2188519 | 1.15(1.11-1.19) | 5.60×10^-16^ | 1.24(1.2-1.29) | <2×10^-16^ | 1.13(1.09-1.17) | 1.40×10^-12^ |
| **Kidney cancer** | | | | | | | |
| PM_2.5_ pollution(μg/m^3^) | 1320/4704865 | 0.98(0.93-1.03) | 0.477 | 1.02(0.97-1.08) | 0.4253 | 0.99(0.94-1.04) | 0.70464 |
| PM_2.5_ pollution (per IQR) | 1320/4704865 | 0.98(0.91-1.04) | 0.477 | 1.03(0.96-1.10) | 0.4253 | 0.99(0.92-1.06) | 0.70464 |
| Low PM_2.5_ pollution^§^ | 709/2508347 | Reference | Reference | Reference | Reference | Reference | Reference |
| High PM_2.5_ pollution^§^ | 611/2196518 | 0.98(0.88-1.1) | 0.766 | 1.06(0.95-1.18) | 0.3094 | 1.00(0.90-1.12) | 0.95537 |

Definition of abbreviations: PM_2.5_ = particulate matter <2.5µm; HR=hazard ratio; CI=confidence interval.

§: Defined by WHO guideline value of PM_2.5_: low (<10 μg/m^3^) and high (>10 μg/m^3^).

Model1: crude model

^*^Model 2: adjusted for age, sex and ethnicity.

^†^Model 3: Model 2+education level, average total household income before tax, physical activity, smoking status, drinking status, BMI, SBP and HbA1c.

**Table S4**

Sequences of qPCR primers.

| Gene | Forward primer | Reverse primer |
| --- | --- | --- |
| Mouse KIM-1  Mouse NGAL  Mouse MCP-1 | CTGCTGCTACTGCTCCTTGT  ACGGACTACAACCAGTTCGC  TTAAAAACCTGGATCGGAACCAA | GCAACCACGCTTAGAGATGC  GGGACAGCTCCTTGGTTCTT  GCATTAGCTTCAGATTTACGGGT |
| Mouse IL-1β | CCTTCCAGGATGAGGACATGA | AACGTCACACACCAGCAGGTT |
| Mouse IL-18 | GACTCTTGCGTCAACTTCAAGG | CAGGCTGTCTTTTGTCAACGA |
| Mouse HO -1 | CGTCACTTCGTCAGAGGCCTGC | TCTGGGGTTTCCCTCGGGGTG |
| Mouse SOD-1  Mouse NOX4 | AACCAGTTGTGTTGTCAGGAC  AGTCAGGTCTGTTTTCTTGCC | CCACCATGTTTCTTAGAGTGAGG  CTCTACTGGATGACTGGAAACC |
| Mouse NLRP3 | ATTACCCGCCCGAGAAAGG | TCGCAGCAAAGATCCACACAG |
| Mouse ASC | TCACAGAAGTGGACGGAGTG | TCATCTTGTCTTGGCTGGTG |
| Mouse Caspase-1 | ACAAGGCACGGGACCTATG | TCCCAGTCAGTCCTGGAAATG |
| Mouse GSDMD | TTAATTGAGGCGGCAGACTT | TGGGCTGGTCCTGTAAAATC |
| Mouse mTOR  Mouse ATG5  Mouse beclin1  Mouse P62 | ACCGGCACACATTTGAAGAAG  TGTGCTTCGAGATGTGTGGTT  GAAACTGGACACGAGCTTCAAGA  AGAAACCCATGGACAGCATC | CTCGTTGAGGATCAGCAAGG  ACCAACGTCAAATAGCTGACTC  ACCATCCTGGCGAGTTTCAATA  GGCCAATAAGATGGGTCTGA |
| Mouse Caspase-3 | GACTGATGAGGAGAATGGCTTG | TGCAAAGGGACTGGATGAAC |
| Mouse BAX | TGAAGACAGGGGCCTTTTTG | AATTCGCCGGAGACACTCG |
| Mouse BCL-2 | GTCGCTACCGTCGTGACTTC | CAGACATGCACCTACCCAGC |
| Mouse β-actin  Human KIM-1  Human NGAL  Human GSDMD  Human Caspase-1  Human NLRP3  Human IL-18  Human mTOR  Human ATG5  Human beclin1  Human LC3B  Human P62  Human β-actin | TTCGTTGCCGGTCCACACCC  TGGCAGATTCTGTAGCTGGTT  CCACCTCAGACCTGATCCCA  GCCTCCACAACTTCCTGACAGATG  CACACCGCCCAGAGCACAAG  AGAGCCCCGTGAGTCCCATTAAG  GCATCAACTTTGTGGCAAT  TCCGAGAGATGAGTCAAGAGG  AAAGATGTGCTTCGAGATGTGT  CCATGCAGGTGAGCTTCGT  AAGGCGCTTACAGCTCAATG  GCACCCCAATGTGATCTGC  GCATGGGTCAGAAGGATTCCT | GCTTTGCACATGCCGGAGCC  AGAGAACATGAGCCTCTATTCCA  CCCCTGGAATTGGTTGTCCTG  GGTCTCCACCTCTGCCCGTAG  TCCCACAAATGCCTTCCCGAATAC  CGCCCAGTCCAACATCATCTTCC  CCGATTTCCTTGGTCAAT  CACCTTCCACTCCTATGAGGC  CACTTTGTCAGTTACCAACGTCA  GAATCTGCGAGAGACACCATC  CTGGGAGGCATAGACCATGT  CGCTACACAAGTCGTAGTCTGG  TCGTCCCAGTTGGTGACGAT |
